# Forest type and stand age co-regulate iron-associated carbon and microbial life-history strategies in red soils

**DOI:** 10.3389/fmicb.2026.1780540

**Published:** 2026-04-30

**Authors:** Fuxing Tan, Renlu Liu, Yian Wang, Zhijun Cao, Houwen Zhang, Li Yin, Genhe He

**Affiliations:** Key Laboratory of Jiangxi Province for Functional Biology and Pollution Control in Red Soil Regions, School of Life Sciences, Jinggangshan University, Ji'an, China

**Keywords:** carbon sequestration, iron oxides, iron-bound organic carbon, microbial life-history strategies, plantation forest, soil organic carbon

## Abstract

Microorganisms are important regulators of soil organic carbon (SOC) accumulation and stabilization, which is modulated by soil minerals, especially iron (Fe) oxides. Here, we analyzed soils from different forest types (coniferous forests, mixed forests, and broad-leaved forests) and stand ages (40-, 20-, and 10-year) in a subtropical acidic red soil region. SOC peaked at 20 years and declined by 40 years, while decreasing with soil depth. Free iron oxides (Fed) content was higher in mixed forests and broad-leaved forests than in coniferous forests, and iron-bound organic carbon (Fe-OC) showed the same pattern among forest types, with Fe-OC dominated by coprecipitation (C:Fe molar ratio >6). Bacterial communities shifted from r- to K-strategists with stand age, with K-strategists (e.g., Chloroflexi) dominating overall and r-strategists (e.g., Gemmatimonadota) enriched in mixed forests. Fungal richness was highest in 40-year broad-leaved forests. Functional predictions suggested potential shifts in microbial functional traits across forest types: coniferous forests stabilize carbon via high-quality litter in oligotrophic conditions; mixed forests showed acidophilic bacteria negatively correlated with Fed, inhibiting Fe-OC dissociation; and broad-leaved forests enhanced Fe-OC coprecipitation via unspecific monooxygenase. Structural equation modeling (SEM) analysis showed that Fed accumulation strongly promoted Fe-OC synthesis. Correlation analysis further indicated that SOC was significantly positively correlated with Fed, carbon fractions, and microbial activity (*p* < 0.05). In conclusion, plantations in the red soil region synergistically enhance soil carbon sequestration potential by regulating iron oxide dynamics and microbial functions, and deep soils, especially 40-year mixed forests, harbor substantial carbon sequestration capacity.

## 1 Introduction

Under the long-term hydrothermal conditions of high-temperature climate and alternating dry-wet seasons in red soil regions, the soils undergoes intense leaching (Zhu et al., 2024). This process leads to the massive migration and loss of base cations, resulting in the formation of acidic red soil characterized by low base saturation and high contents of free iron (Fe) and aluminum (Al) oxides (Huang et al., 2010; Li et al., 2025). Such Fe-Al-rich conditions provide a distinctive geochemical environment for soil organic carbon (SOC) stability. Soil minerals protect SOC from microbial degradation through adsorption, occlusion, and aggregation, among which Fe oxides are the key minerals for forming organomineral complexes (Eusterhues et al., 2005). Fe oxides stabilize SOC mainly through adsorption and coprecipitation (Shabtai et al., 2023). The dominant mechanism can be inferred from the carbon-to-iron molar ratio (C:Fe) of iron-bound SOC: ratios < 1 indicate adsorption, while ratios >6 suggest co-precipitation, wherein SOC and dissolved Fe form amorphous organo-mineral complexes (Wang et al., 2017). Free iron oxides (Fed), as a highly reactive Fe pool in soils (Huang et al., 2025), dominate SOC stabilization via coprecipitation in acidic red soil environments, thereby effectively suppressing microbial enzymatic decomposition of SOC. Meanwhile, Fe oxides exert a dual control on SOC: they act as terminal electron acceptors for microbial mineralization of SOC, enabling microbially mediated electron transfer; and promoting soil aggregation, thereby physically protecting SOC from microbial degradation (Jiang et al., 2021; Liu et al., 2019). Investigating the role of Fe oxides in SOC stabilization in red soil ecosystems is therefore essential for understanding SOC sequestration in plantation forests.

Soil microorganisms are critical intermediaries between SOC inputs and outputs (Bhattacharyya et al., 2022), driving soil biogeochemical cycles and maintaining forest ecosystem functioning (Kumar et al., 2022). According to the Microbial Carbon Pump (MCP) theory, microorganisms assimilate plant-derived organic compounds into biomass, and their residues are subsequently stabilized via the “entombing effect” through association with soil minerals (Liang et al., 2017). The composition, biomass, and activity of soil microbial communities directly regulate SOC mineralization and immobilization processes (Shen et al., 2023), ultimately determining the magnitude and persistence of SOC storage (Kästner and Miltner, 2018). Numerous studies have reported significant correlations between SOC stability and the structural and functional diversity of soil microbial communities (Pajares and Bohannan, 2016; Qu et al., 2020; Wang C. et al., 2021). Microbial community composition and functional attributes are shaped by differentiated resource acquisition strategies, growth rates, metabolic rates, and environmental tolerance across various taxa and soil environments (Cao et al., 2025; Malik et al., 2020; Zhu et al., 2022). Microbial life-history strategies offer a tractable framework to link microbial identity with ecological functional groups based on morphological, physiological, or life-history traits (Fierer et al., 2007; Zhang Y. et al., 2024). r/K-selection strategies have been shown to exert strong control over decomposition rates, transformation pathways, and long-term stability of SOC (Wang et al., 2024). K-strategist microorganisms efficiently utilize labile SOC and typically dominate in carbon-limited/oligotrophic environments, whereas r-strategists preferentially exploit readily decomposable SOC, exhibiting rapid growth but lower resource use efficiency (Liu et al., 2025). Vegetation restoration and improved management practices that shift microbial communities from r- to K-strategists can enhance SOC sequestration potential by over 30% (Zhu et al., 2025). Therefore, understanding the composition of soil microbial communities and the regulatory factors governing their life strategies is crucial for quantifying and managing carbon sinks in forest ecosystems. However, soil acidification in red soil regions suppresses microbial growth and activity, which in turn limits research on the relationship between microbial life strategy shifts and SOC stability.

The interaction between iron oxides and microorganisms is central to regulating the balance between soil organic carbon sequestration and mineralization. Negatively charged functional groups on microbial surfaces can adsorb iron oxides and promote the formation of bio-mineral aggregates. The resulting hierarchical aggregate structure features low oxygen availability and impeded enzyme diffusion, which can reduce the decomposition rate of encapsulated organic carbon by 50%−80%, thereby favoring the long-term sequestration of soil organic carbon. For instance, K-strategists (e.g., *Steroidobacter* and *Cryptococcus*) become significantly enriched in agricultural root deposits. Their hyphae penetrate mineral barriers and secrete metabolites that promote Fe oxide stabilization and aggregate development (Jeewani et al., 2020). In wetland systems, co-precipitation of Fe-plaque communities from plant roots with Fe oxides significantly enhances SOC sequestration capacity (Duan et al., 2020). Amorphous Fe oxides could inhibit the transformation of SOC into complex aromatic structures by reducing microbial utilization frequency, thereby forming a stable carbon pool dominated by aliphatic carbon (Tian and Lu, 2023).

Subtropical regions represent the primary distribution areas of acidic red soils in China. The prevailing climate, characterized by high temperature, humidity, and distinct alternation between dry and wet seasons, promotes iron oxidation in the soil, leading to the formation of Fe oxide-rich red soils (Chen et al., 2014). The structure and function of microbial communities, in turn, influence Fe oxide-SOC interactions. In paddy soils, a competitive relationship exists between Fe(III)-reducing bacteria and methanogens, whereby the activity of Fe(III)-reducing bacteria can reduce methane emissions from rice fields (Wang J. et al., 2021). However, most existing studies on Fe oxide-microbe interactions have been confined to wetland and agricultural systems, leaving a critical gap in our understanding of subtropical red soil plantation ecosystems. Previous studies have typically examined only single factors or single dimensions, which has prevented a full understanding of how temporal dynamics, spatial heterogeneity, and vegetation complexity jointly regulate the synergistic stabilization of soil organic matter by iron and microorganisms in subtropical red soil ecosystems. Addressing these gaps is essential for predicting the long-term carbon sequestration potential of afforestation practices in subtropical regions.

The structure and diversity of soil microbial communities are significantly shaped by aboveground vegetation types (Bever et al., 2012) and soil depth (Hao et al., 2021). Compared to monoculture plantations, mixed forests generally exhibit higher plant diversity and biomass productivity (Lei et al., 2023; Ming et al., 2018). Combined with the microbial carbon pump theory, the microbial life strategy framework, and the biogeochemical processes of iron redox, we propose the following hypotheses: (1) during forest development, r-strategist microbes in young stands gradually shift to K-strategist microbes in mature stands; (2) the biological mechanism dominated by microbial life-history strategies prevails in topsoil, while the abiotic control mechanism protected by iron oxides dominates in subsoil; and (3) compared with monoculture plantations, mixed plantations can more effectively promote the formation and accumulation of active iron-bound organic carbon fractions by altering microbial life-history strategies. To test these hypotheses, our experimental design encompassed three plantation types with stand-age chrono sequences of 40, 20, and 10 years to track the temporal dynamics soil carbon pools and Fe-carbon complexes. Amplicon sequencing was utilized to characterize microbial community reassembly and life-strategy adaptations, thereby evaluating the synergistic roles of Fe oxides and microbiota in SOC stabilization. We acknowledge that the space-for-time substitution approach used here carries inherent assumptions; however, it remains a valuable tool for inferring decadal-scale ecological processes where long-term monitoring data are unavailable. The specific objectives were: (1) to analyze the dynamics of SOC and its fractions across different plantation types; (2) to characterize shifts in soil microbial community diversity and life strategies under varying plantation regimes; and (3) to elucidate the stabilization mechanisms of SOC co-mediated by microbial communities and Fe oxides.

## 2 Materials and methods

### 2.1 Site description and soil sampling

The study area (26°4′39″N, 115°3′33″E) is located at the Qianyanzhou Ecological Station in Jiangxi Province, southeastern China (Supplementary Figure S1). This region experiences a typical East Asian subtropical monsoon climate, with mean annual precipitation of 1,489 mm and a mean annual temperature of 17.9 °C, primarily occurring between March and June. Seasonal droughts frequently occur from July to October due to scarce rainfall and elevated temperatures (Yang et al., 2015). The soils are classified as typical Ultisols (USDA Soil Taxonomy), formed from weathering of red sandstone and sandy conglomerate derived from Quaternary red clay deposits (Xia et al., 2025). The plantation ecosystems primarily consist of coniferous forests, broadleaved forests, and mixed conifer-broadleaf forests. Coniferous forests are dominated by *Pinus massoniana, Pinus elliottii*, and *Cunninghamia lanceolata*, while broadleaved forests are principally composed of *Cinnamomum camphora, Eucalyptus* spp., *Schima superba*, and *Liquidambar formosana*. Mixed conifer-broadleaf forests are predominantly composed of *Cunninghamia lanceolata* and *Liquidambar formosana*. The understory vegetation includes *Diplazium esculentum, Parthenocissus quinquefolia, Spatholobus suberectus, Lophatherum gracile, Adiantum capillus-veneris, Camellia oleifera, Ilex pubescens*, and *Loropetalum chinensis*.

Three plantation types were selected: coniferous plantation (CF), mixed conifer-broadleaf plantation (MF), and broadleaved plantation (BF). Each type included stands aged approximately 40, 20, and 10 years. Three 20 × 20 m standard plots were randomly designated for each stand age, with an interval of 100 m between plots. Before sampling, the plots were inspected, and relevant information such as understory vegetation was recorded. A total of 27 plots were established across the nine groups of planted forests (3 stand ages × 3 forest types × 3 replicate plots). For each plot, soil samples were collected from depths of 0–20, 20–60, and 60–100 cm using the five-point sampling method. Soil samples from the same soil layer of 15 points across the three replicate plots of each planted forest type were mixed into a composite sample, resulting in a total of 81 soil samples (3 stand ages × 3 forest types × 3 soil layers × 3 replicates). Each composite sample was divided into three subsamples: one stored at 4 °C for determining soil moisture content, microbial biomass carbon, and dissolved SOC within 1 week; another air-dried after removing visible litter, roots, and stones, then sieved through a 2-mm mesh for analyzing physicochemical properties, Fe oxides, and SOC fractions; and the third preserved at −80 °C for microbial community analysis. A chronosequence sampling approach was employed, leveraging the space-for-time substitution hypothesis to investigate temporal dynamics of soil microbial functional stability across stand age gradients.

### 2.2 Soil physicochemical analyses

Soil moisture content (SMC) was determined by oven-drying at 105 °C to constant weight. Soil pH was measured in a 1:2.5 (w:v) air-dried soil-to-water suspension using a glass electrode pH meter. Total nitrogen (TN) was determined using a fully automated Kjeldahl nitrogen analyzer. SOC was measured by the potassium dichromate (K_2_Cr_2_O_7_) oxidation method (Nelson and Sommers, 1982). Soil microbial biomass carbon (MBC) was analyzed using the chloroform fumigation-extraction method, with unfumigated soil samples as controls (Mo et al., 2008). Soil dissolved organic carbon (DOC) was extracted with potassium sulfate solution and quantified using a TOC analyzer (Multi N/C 3100, Analytik Jena AG, Germany). Readily oxidizable organic carbon (ROC) was determined using the 333 mmol L^−1^ potassium permanganate (KMnO_4_) oxidation method. Blank controls without soil were included. The oxidized carbon content was quantified spectrophotometrically at 565 nm (Blair et al., 1995). Particulate organic carbon (POC, 53–2000 μm) and mineral-associated organic carbon (MAOC, < 53 μm) were determined using the sodium hexametaphosphate wet-sieving method (Cambardella and Elliott, 1992).

### 2.3 Analysis of Fe oxides

Free iron oxides (Fed) were extracted using the dithionite-citrate-bicarbonate (DCB) method (Mehra, 1958). Amorphous iron oxides (Feo) were extracted using the ammonium oxalate method (Schwertmann, 1964). Complexed iron oxides (Fep) were extracted using sodium pyrophosphate solution (Mikutta et al., 2006). Crystalline free Fe (Fec) were calculated as the difference between Fed and Feo.

*The iron activation degree* (%) = *Feo*/*Fed* × 100%

*The iron complexation index* (%) = *Fep*/*Fed* × 100%

Iron-bound SOC content was determined synchronously with the DCB extraction procedure (Lalonde et al., 2012). Soil samples were mixed with a buffer solution (0.27 M trisodium citrate and 0.11 M sodium bicarbonate). Control experiments used a mixed sodium chloride solution (0.11 M sodium bicarbonate and 1.60 M sodium chloride) to account for organically released carbon during heating. Mixtures were centrifuged, rinsed, freeze-dried, and residues analyzed for SOC content using the K_2_Cr_2_O_7_ oxidation method. The percentage of iron-bound SOC (fFe-OC) and the OC:Fe molar ratio were calculated as follows:

*fFe* − *OC* = *Fe* − *OC*/*SOC* × 100%

*Fe* − *OC* = *OC* < *uscore* > *NaCl* − *OC* < *uscore* > *DCB*

*C*:*Fe molar ratio* = (*Fe* − *OC*/*M* < *uscore* > *c*)/(*m* < *uscore* > *Fed*/*M* < *uscore* > *Fe*)

where M_c and M_Fe are the molar masses of C and Fe, respectively. C:Fe molar ratios <1 indicate adsorption, 1–6 complexation, and >6 co-precipitation (Wang et al., 2017).

### 2.4 DNA extraction and high-throughput sequencing

Soil DNA was extracted from 0.5 g samples using a FastDNA Kit (MP Biomedicals, Solon, OH, USA). DNA was dissolved in 50 μl TE buffer and quantified using a Nanodrop 2000 (Thermo Scientific, Waltham, MA, USA). The bacterial 16S rRNA V4–V5 regions were amplified with primers 515F and 806R (Bates et al., 2011). The fungal ITS1 region was amplified with primers ITS1F and ITS2 (Borneman and Hartin, 2000). After three amplification rounds, products were pooled and purified using Agincourt Ampure XP beads. All amplicons were combined at equimolar concentrations (20 ng·μl^−1^) and sequenced on the Illumina HiSeq 2000 platform (250 bp paired-end). Amplification and sequencing were conducted by Major Bio (Shanghai, China).

Raw reads were processed using a standardized pipeline: demultiplexing, quality filtering with Trimmomatic (v0.39), and assembly with FLASH (v1.2.11). Taxonomic classification was assigned using the RDP classifier against the SILVA 138 (16S) and UNITE 8.0 (ITS) databases with a confidence threshold of 0.7 (Chen et al., 2017). Sequences were clustered into OTUs at 97% similarity using UPARSE (v7.1) within USEARCH (v11), and chimeras were removed with UNOISE3. Alpha diversity indices (Richness, Shannon) were calculated using the vegan package in R (v4.1.0). Beta diversity was assessed based on Bray–Curtis distances and visualized via PCoA. PERMANOVA (adonis2, 999 permutations) tested the effects of forest type, age, and depth on community composition. RDA explored relationships between microbial communities and environmental variables. SEM was performed using the lavaan package, with model fit evaluated using χ^2^-test (*p* > 0.05), CFI (>0.95), and RMSEA (<0.06). Functional profiles of prokaryotic communities were predicted using PICRUSt2 with the MetaCyc database. Fungal functional guilds were inferred using FUNGuild. To distinguish the life-history strategies of microorganisms, this study adopted a classification method based on the copy number of the 16S rRNA gene. We followed the protocol established by (Hu et al. 2023), which calculates the average copy number of each taxon by querying the rrnDB database. Based on this, major microbial phyla and genera were defined as either r-strategists (high copy number, tending to grow rapidly) or K-strategists (low copy number, tending to have high competitive efficiency).

### 2.5 Statistical analyses

Analysis of variance (ANOVA) was used to examine differences in soil components across layers. General Linear Models (GLM) and Duncan's multiple range test analyzed the effects of forest type, stand age, soil depth, and their interactions on SOC pools. Pearson correlation and stepwise regression explored relationships between SOC pools and soil properties. Variables were log-transformed to meet normality assumptions. All statistical analyses were performed using IBM SPSS Statistics 22.0 and Microsoft Excel 2016. PCoA and PERMANOVA (999 permutations) based on Bray–Curtis distances were performed using the vegan package. Results were visualized with ggplot2. RDA examined relationships between microbial communities and environmental factors. Linear regression and Pearson correlation assessed linkages between soil variables and microbial community characteristics. PCA evaluated associations of SOC accumulation with soil properties, Fe oxides, and microbial attributes. Functional profiles were predicted using PICRUSt2 (Douglas et al., 2019). Correlation analyses used the psych package, and heatmaps were generated with pheatmap. IBM SPSS Amos 25.0 (Amos Development Corporation, Meadville, PA, United States) was used to establish the structural equation model (SEM) among plantation forest age, bacterial diversity, fungal diversity, Fed, Fe-OC and SOC. An SEM theoretical model between independent and dependent variables was established using AMOS software, and the model was further optimized based on the calculated results of the theoretical model. The chi-square test (*p* > 0.05) indicated a good fit between the model and the data.

## 3 Results

### 3.1 Soil properties and Fe-carbon coupling

**Figure 1:**
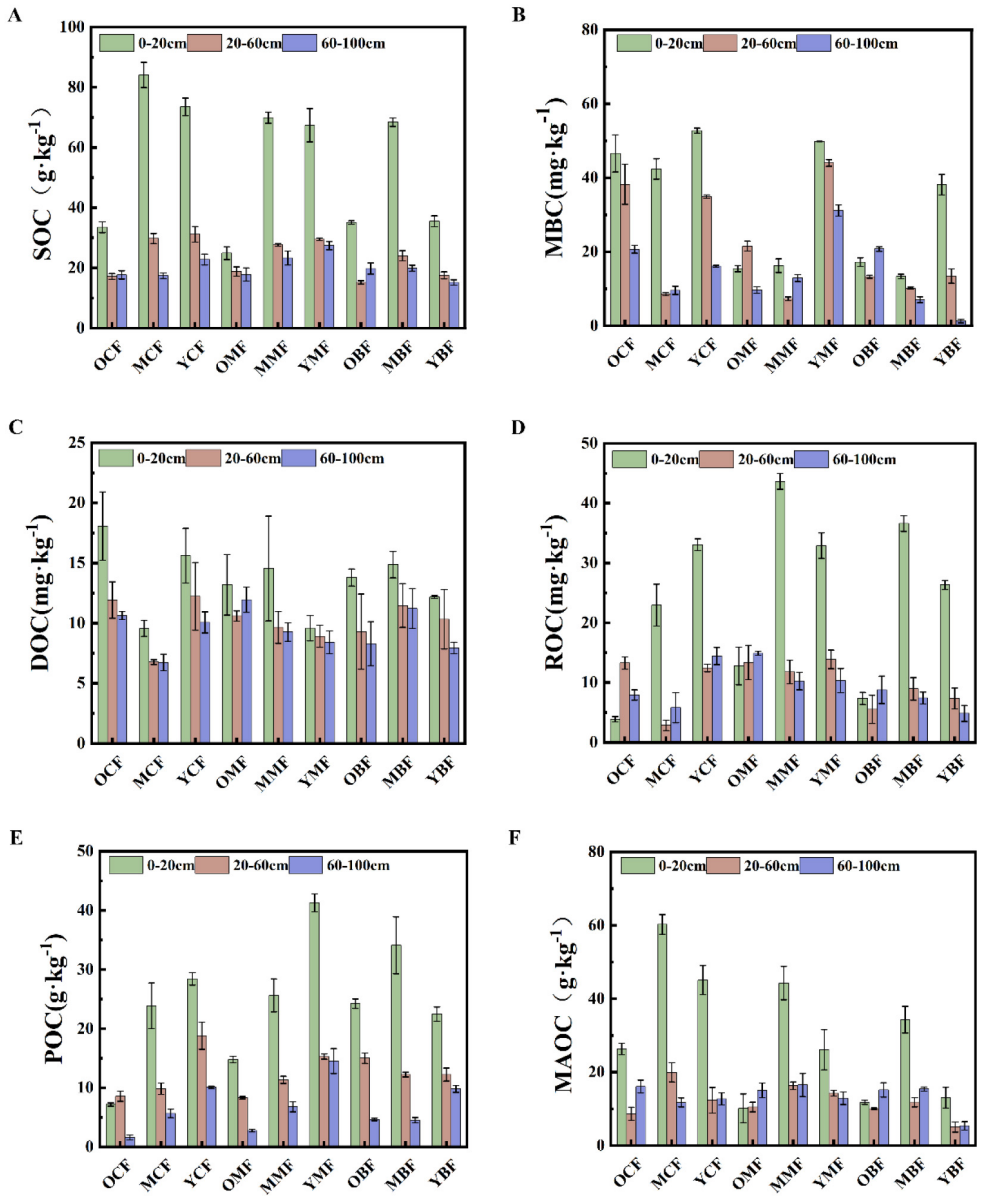
Contents of SOC and its components in plantations at different forest ages and in different soil layers. SOC: soil organic carbon **(A)**; MBC: microbial biomass organic carbon **(B)**; DOC: soil dissolved organic carbon **(C)**; ROC: readily oxidizable carbon **(D)**; POC: particulate organic carbon **(E)**; MAOC: mineral-associated organic carbon **(F)**. OCF, 40 year coniferous forest; MCF, 20 year coniferous forest; YCF, 10 year coniferous forest; OMF, 40 year mixed forest; MMF, 20 year mixed forest; YMF, 10 year mixed forest; OBF, 40 year broad-leaved forest; MBF, 20 year broad-leaved forest; YBF, 10 year broad-leaved forest.

Significant differences were observed in SOC and its fractions across plantation stands of different ages (Figure 1). Total nitrogen (TN) and SOC content decreased with increasing stand age and soil depth, showing a gradient pattern: coniferous forests > mixed forests > broadleaved forests (Supplementary Table S1). The 20-year coniferous plantation exhibited the highest surface SOC content (84.1 g·kg), significantly greater than 40-year (*p* < 0.01) and 10-year (*p* < 0.05) stands. In the 40-year-old mixed forests (OMF) and broad-leaved forests (OBF), the mineral-associated organic carbon (MAOC) content in the deep layers (60–100 cm) was significantly higher than that in the topsoil, reflecting the carbon sequestration potential of the deep soil. In contrast, the 10-year stand had relatively high surface SOC (67.4 g·kg^−1^) with a gradual decline with depth. DOC content increased markedly, but did not differ significantly among forest types. Mineral-associated organic carbon (MAOC) peaked at 20 years, while particulate organic carbon (POC), microbial biomass carbon (MBC), and readily oxidizable carbon (ROC) decreased with stand age. ROC reached maximum values at 20 years in mixed and broadleaved forests, but at 10 years in coniferous forests. These results indicate that SOC and its fractions in subtropical plantations were generally higher in 10-and 20-year stands than in 40-year stands (Figure 1). Soil pH ranged from 4.28 to 5.23 and was lowest in deep layers of 20-year coniferous plantations. Soil water content (SWC) was higher in surface layers, with all horizons in 40-year broadleaved plantations maintaining SWC values of over 22% (Supplementary Table S1).

**Figure 2:**
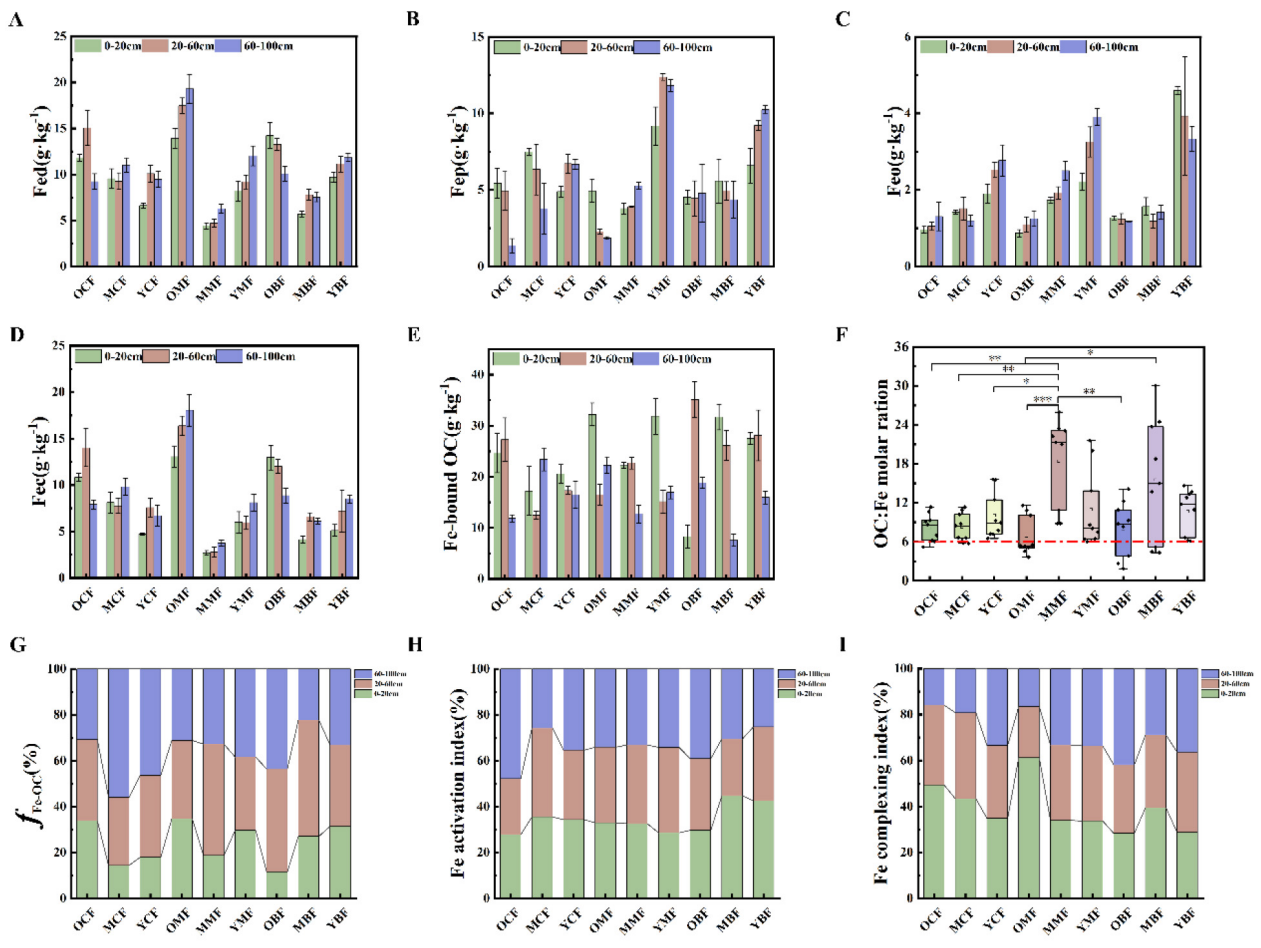
Changes in soil Fe oxides and their related indices in plantations at different forest ages and in different soil layers. Fed: free iron oxides **(A)**; Fep: complexed iron oxides **(B)**; Feo: amorphous iron oxides **(C)**; Fec: crystalline free Fe **(D)**; Fe-bound organic carbon (Fe–OC) **(E)**; molar ratio of Fe–OC to total free Fe **(F)**; *f* Fe-OC (ratio of Fe–OC to SOC) **(G)** Fe activation index: Feo:Fed **(H)** and Fe complexing index: Fep:Fed **(I)**. OCF, 40 year coniferous forest; MCF, 20 year coniferous forest; YCF, 10 year coniferous forest; OMF, 40 year mixed forest; MMF, 20 year mixed forest; YMF, 10 year mixed forest; OBF, 40 year broad-leaved forest; MBF, 20 year broad-leaved forest; YBF, 10 year broad-leaved forest. *, **, *** indicate *p* < 0.05, *p* < 0.01, and *p* < 0.001, respectively.

The distribution of Fe oxide forms varied significantly with soil depth (*p* < 0.001). Across all layers, total content followed: Fed > Fec > Fep > Feo (Figures 2A–D). Fed exhibited a V-shaped pattern (initial decrease and then increase) in mixed and broadleaved forests but decreased monotonically in coniferous forests (Figure 2A). Both crystalline free Fe and Feo decreased with stand age, with the highest values observed at 10 years (Figures 2B–D). Fe-OC increased overall but showed vertical heterogeneity as it was enriched in middle layers in broadleaved forests, while mixed and coniferous forests displayed distinct depth-dependent patterns (Figures 2E, G). The iron activation degree and complexation index decreased with stand age and soil depth (Figures 2H, I). These findings suggest that mixed and broadleaved forests enhance iron-carbon coupling by modulating Fed dynamics, while coniferous forests exhibit pronounced Fe oxide aging. Analysis of C:Fe ratios revealed that only minor portions of Fe-OC in 40-year broadleaved plantations were formed via adsorption, while coprecipitation was the dominant binding mechanism in most plantations (Figure 2F).

### 3.2 Soil microbial community characteristics and life strategies

**Figure 3:**
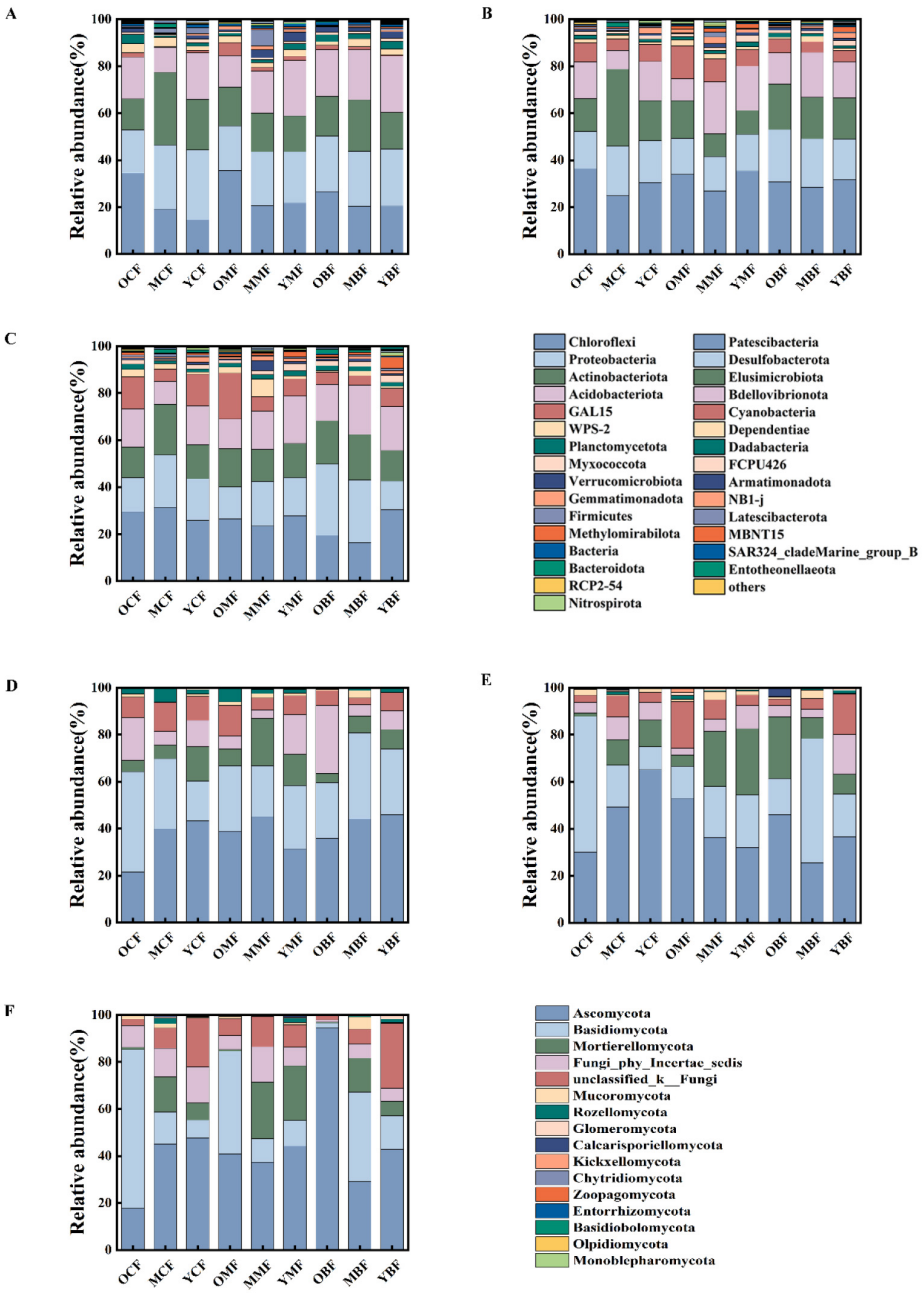
Changes in the abundances of soil bacteria **(A–C)** and fungi **(D–F)** in plantations at different forest ages and in different soil layers. **(A, D)** 0–20 cm; **(B, E)** 20–60 cm; **(C, F)** 60–100 cm. OCF, 40 year coniferous forest; MCF, 20 year coniferous forest; YCF, 10 year coniferous forest; OMF, 40 year mixed forest; MMF, 20 year mixed forest; YMF, 10 year mixed forest; OBF, 40 year broad-leaved forest; MBF, 20 year broad-leaved forest; YBF, 10 year broad-leaved forest.

The dominant bacterial phyla across all samples were *Chloroflexi, Proteobacteria, Actinobacteria, Acidobacteria*, and *Patescibacteria* (Figures 3A–C). They were classified as K-strategists, except *Proteobacteria*. *Gemmatimonadota* and *Firmicutes*, abundant in mixed forests, were identified as r-strategists. *Firmicutes* peaked in 20-year mixed plantations (7.09%) but remained below 1% in other types. Actinobacteria peaked in 20-year coniferous plantations (32.8%), and were also prominent but reached maxima in 40-year mixed (SOC: 69.89 g·kg^−1^) and broadleaved forests (SOC: 68.4 g·kg^−1^). Despite overall decline, with stand age, r-strategists (*Gemmatimonadota* and *Firmicutes*) remained predominant in mixed forests and 10-year plantations.

Fungal communities were dominated by *Ascomycota, Basidiomycota*, and *Mortierellomycota* (Figures 3D–F). In coniferous plantations, *Ascomycota* peaked in the middle layers of 10-year stands (65.2%), while *Basidiomycota* peaked in deep layers of 40-year stands (67.4%). In mixed and broadleaved forests, *Ascomycota* showed an inverse relationship to *Basidiomycota* and *Mortierellomycota*, though no consistent age-related trend was evident. *Mortierellomycota* peaked (28.25%) in mid-layers of 10-year mixed forests. *Ascomycota* were classified as r-strategists, while *Basidiomycota* were classified as K-strategists.

**Figure 4:**
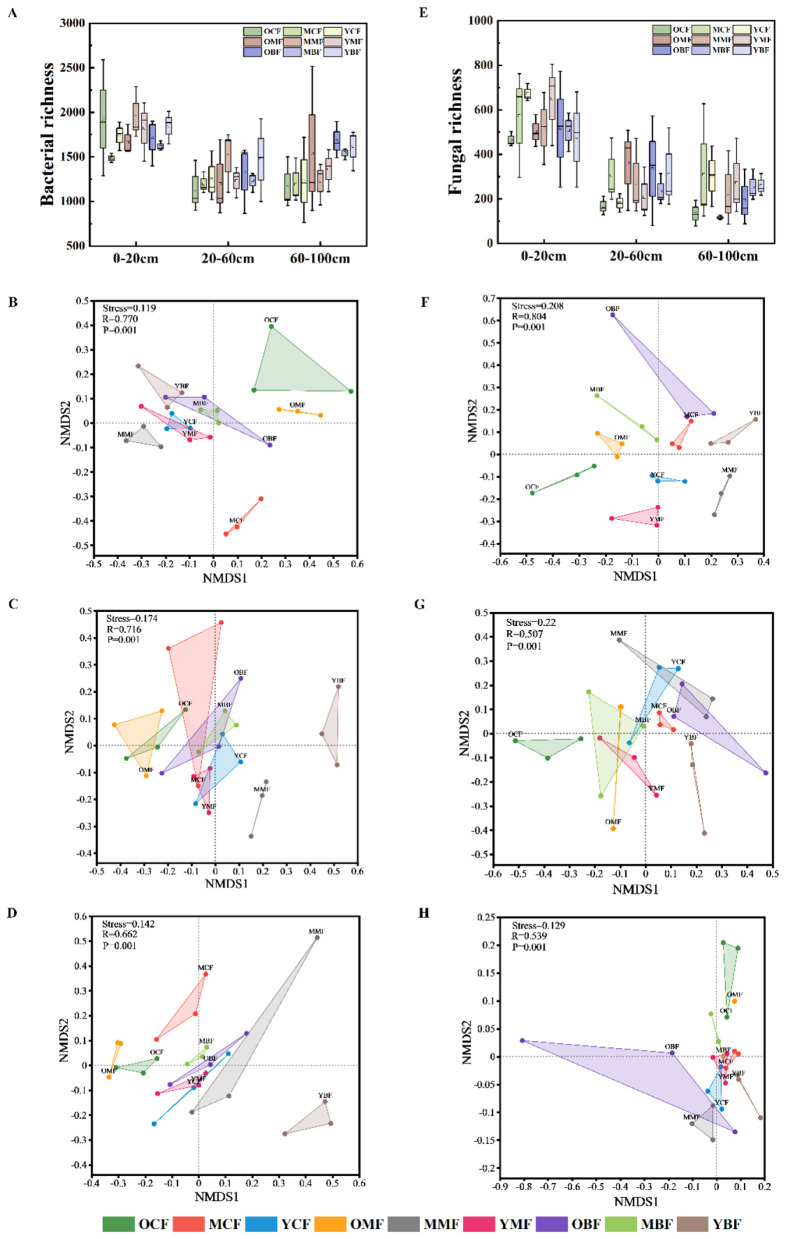
Non-metric multidimensional scaling (NMDS) ordination plots of the richness and communities of soil bacteria **(A–D)** and fungi **(E–H)** at different forest ages and in different soil layers. **(B, E)** 0–20 cm; **(C, G)** 20–60 cm; **(D, H)** 60–100 cm. OCF, 40 year coniferous forest; MCF, 20 year coniferous forest; YCF, 10 year coniferous forest; OMF, 40 year mixed forest; MMF, 20 year mixed forest; YMF, 10 year mixed forest; OBF, 40 year broad-leaved forest; MBF, 20 year broad-leaved forest; YBF, 10 year broad-leaved forest.

Bacterial and fungal richness decreased with soil depth, correlating with higher organic matter and biological activity in surface layers. Bacterial richness showed U-shaped patterns in coniferous and broadleaved forests (40-year ≈ 10-year > 20-year), while it peaked at 20 years in mixed forests (Figure 4A). Fungal richness exhibited significant forest type × age interactions, being highest in 40-year broadleaved forests but dominated by coniferous plantations at 20 years (Figure 4E). Non-metric multidimensional scaling (NMDS) revealed that soil depth, forest type, and stand age collectively drove microbial community distribution and diversity (Figure 4). In surface layers, bacterial and fungal communities showed widely dispersed distributions with significant variations across ages and types (Figures 4F–H). In contrast, fungal communities in 40-year coniferous forests were clearly separated across all depths. Middle and deep layers showed more convergent microbial distributions, becoming increasingly homogeneous across ages and forest types. Bacterial communities in coniferous and mixed forests varied pronouncedly with age and depth, while broadleaved forests exhibited distinct community patterns only at 10 years (Figures 4B–D).

### 3.3 Soil microbial metabolic function prediction

**Figure 5:**
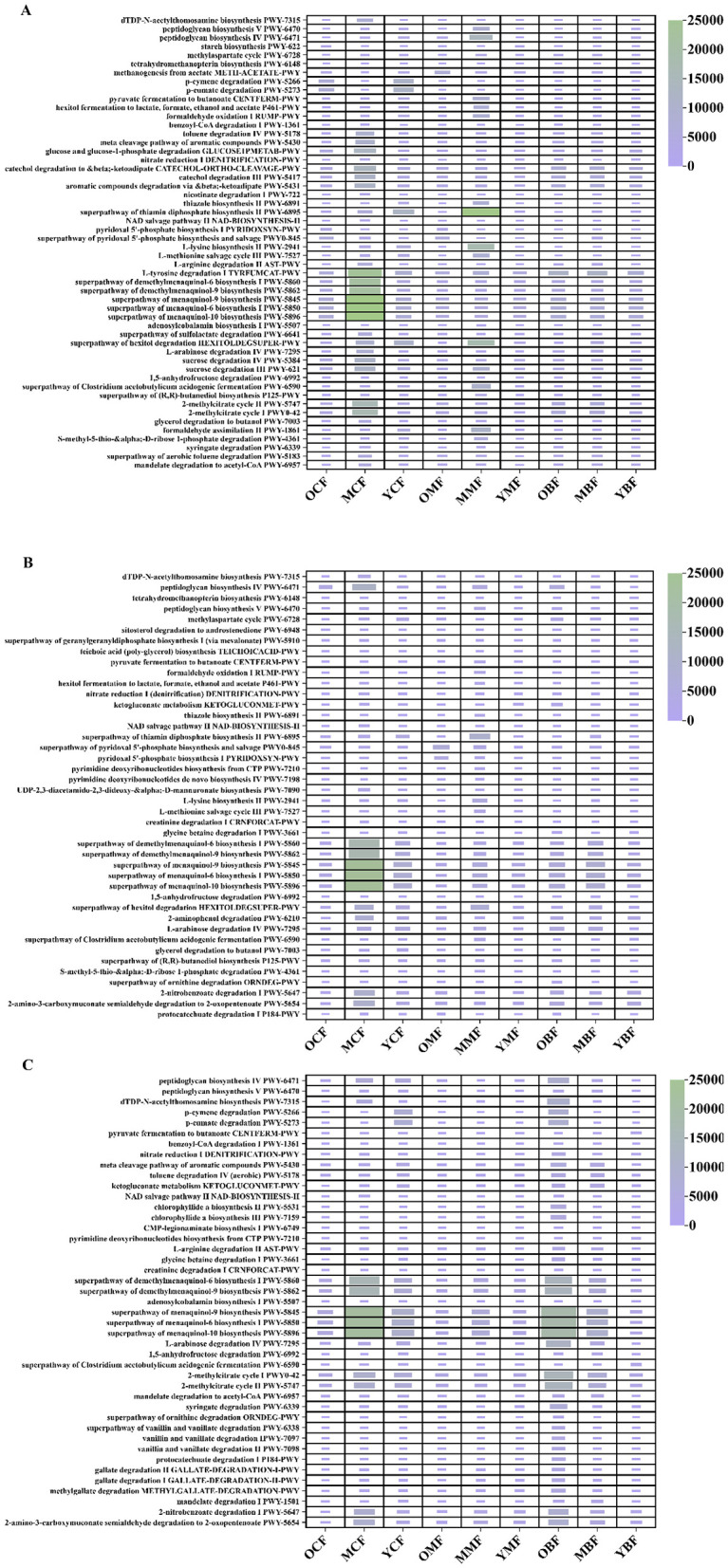
Functional potentials of soil bacteria communities under different forest ages and soil layer depths: **(A–C)** Relative abundances of metabolic pathways predicted by PICRUSt2 based on the MetaCyc database. **(A)** 0–20 cm; **(B)** 20–60 cm; **(C)** 60–100 cm. OCF, 40 year coniferous forest; MCF, 20 year coniferous forest; YCF, 10 year coniferous forest; OMF, 40 year mixed forest; MMF, 20 year mixed forest; YMF, 10 year mixed forest; OBF, 40 year broad-leaved forest; MBF, 20 year broad-leaved forest; YBF, 10 year broad-leaved forest.

PICRUSt2 predictions based on 16S rRNA amplicon sequencing data indicated that the highest bacterial KEGG Orthology (KO) abundances were associated with core growth and metabolic functions (Supplementary Figures S2A–C). Functional gene prediction revealed that chemoheterotrophy, aerobic chemoheterotrophy, and cellulolysis were the most abundant metabolic categories across all soils (Supplementary Figures S3A–C). Notably, 20-year coniferous forests showed elevated predicted abundances in menaquinone/naphthoquinone biosynthetic pathways (MetaCyc: PWY-5860, PWY-5862, PWY-5850, PWY-5845, PWY-5896) compared to other types (ANOVA, *p* < 0.05; Figure 5A). Predicted abundances of pathways related to biosynthesis and cofactor generation (PWY-6895), nitrogen fixation (PWY-2941), polyol degradation (PWY-HEXITOLDEGSUPER), and peptidoglycan synthesis (PWY-6471, PWY-6470) were higher in mixed forests than in monocultures (Figures 5A–C), suggesting that mixed forests more effectively support microbial functions related to nitrogen fixation, complex carbon utilization, and cellular structure synthesis. In deep soil layers (60–100 cm), 40-year broadleaved forests exhibited unique metabolic profiles, with markedly enriched menaquinone/naphthoquinone coenzyme synthesis pathways were significantly elevated (Figure 5C). Concurrently, pathways related to rhizobial nitrogen fixation (PWY-AST), chlorophyll synthesis (PWY-5531, PWY-7159), specialized lipid metabolism (PWY-6339, PWY-1501), and aromatic compound degradation (PWY-5624) were markedly enrichced (Figure 5C).

PICRUSt2 predictions for fungi revealed that key enzymes associated whith SOC stabilization, including β-glucosidase, glucan 1,4-α-glucosidase, and chitinase, peaked in surface soils (0–20 cm) of 40-year mixed plantations and increased with stand age (Supplementary Figure S4A). In coniferous and broadleaved forests, these enzymes reached peak abundances at 20 years, followed by declines. The abundance dynamics of iron-redox coupled enzymes, including NADPH:quinone reductase (NQR) and unspecific monooxygenase (UMO), were closely linked to iron availability: NQR increased steadily with stand age in mixed forests, potentially reflecting enhanced iron reduction, while UMO was enriched at 40 years, consistent with elevated iron oxidation demand during late successional stages (Supplementary Figures S3B, C). Fungal functional guilds in surface soils were dominated by Ectomycorrhizal, Soil Saprotroph, Endophyte-Soil Saprotroph, and Plant Pathogen categories (Supplementary Figure S5). Metabolic pathway analysis showed that energy metabolism (PWY-7279, PWY-7111, PWY-5690, PWY-3781) and lipid metabolism (PWY-7288, PWY-7007, PWY-6351, PWY-5994) had the highest relative abundances across all forests and layers, sustaining fungal growth and carbon transformation (Supplementary Figure S6).

### 3.4 Linkages among microbial communities, Fe oxides, and environmental factors

**Figure 6:**
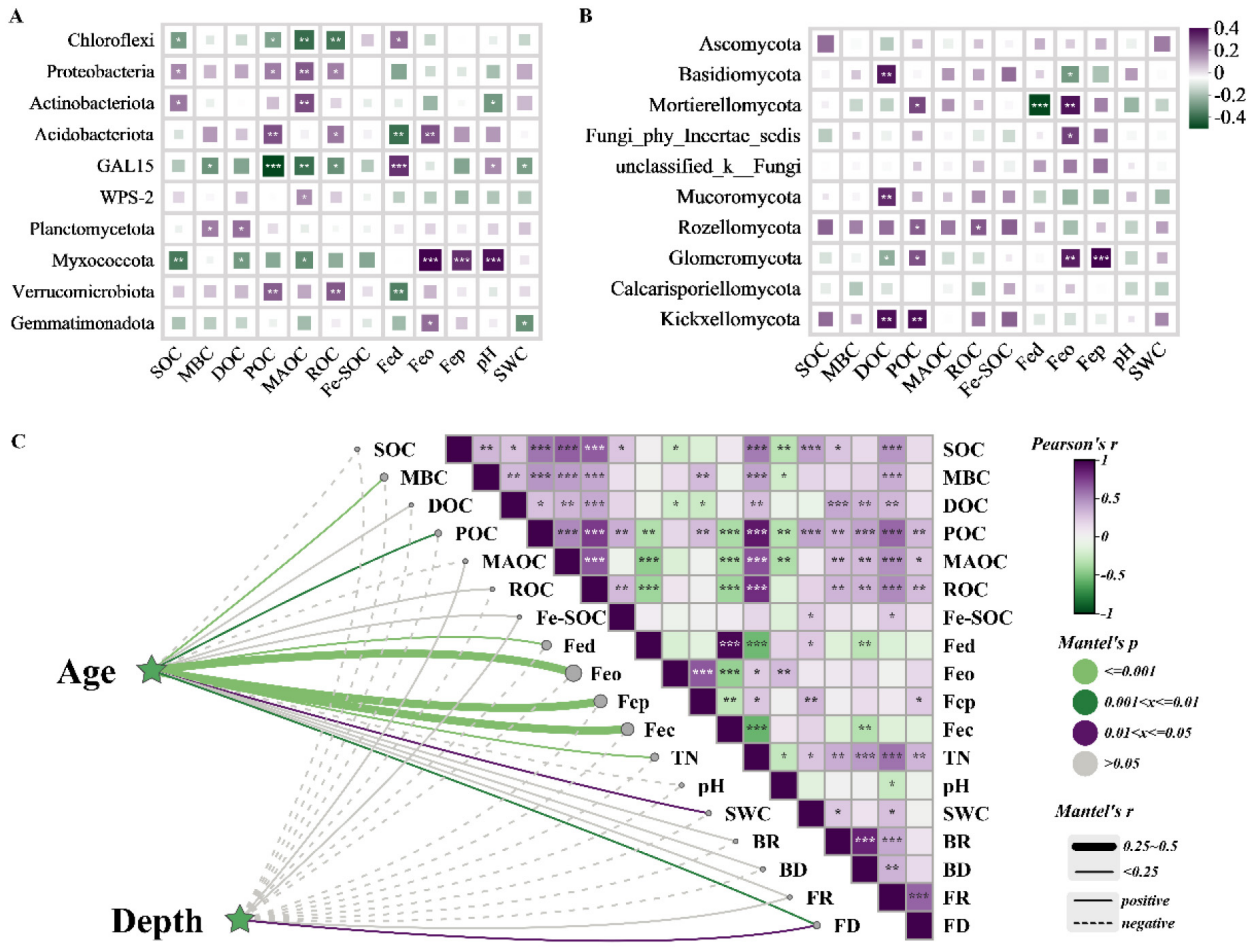
Analysis of the correlation between the main dominant phyla of bacteria **(A)** and fungi **(B)** and environmental factors based on the Pearson coefficient. Mantel test on soil texture, Fe oxides, SOC and its components with soil layer and forest age **(C)**. Fed, free iron oxides; Fep, complexed iron oxides; Feo, amorphous iron oxides; Fec, crystalline free Fe; Fe-SOC, Fe-bound organic carbon; SOC, soil organic carbon; MBC, microbial biomass organic carbon; DOC, soil dissolved organic carbon; ROC, readily oxidizable carbon; POC, particulate organic carbon; MAOC, min-eral-associated organic carbon; TN, total nitrogen; SWC, soil water content; BR, bacterial richness; FR, fungal richness; BD, bacterial diversity; FD, fungal diversity. *, **, *** indicate *p* < 0.05, *p* < 0.01, and *p* < 0.001, respectively.

Pearson correlation analysis revealed that SOC content was the key factor influencing microbial community compositions (Mantel test, *r* = 0.45, *p* = 0.001), while soil pH and moisture also significantly affected specific microbial phyla (Figures 6A, B). Bacterial phyla Chloroflexi and Proteobacteria, and fungal phyla *Ascomycota* and *Basidiomycota*, were more abundant in organic matter-rich environments, consistent with their central roles in decomposition and nutrient cycling (Figures 6A, B). Fungal communities exhibited higher diversity in response to environmental factors, reflecting greater functional and ecological adaptability. *Acidobacteriota, Myxococcota, Mortierellomycota*, and *Glomeromycota* showed strong correlations with Fe oxide content. Significant positive correlations (*p* < 0.05) were observed between SOC and MBC, DOC, POC, MAOC, ROC, Fe-SOC, TN, SWC, BR, and FR (Figure 6C). Correlations between Fe-SOC and Fed/Fep indicate crucial roles of Fe oxides in SOC stabilization. Stand age positively affected SWC (*p* < 0.05), but negatively affected MBC, POC, Fed, Feo, Fep, Feh, FD, and TN (*p* < 0.05; Figure 6C).

**Figure 7:**
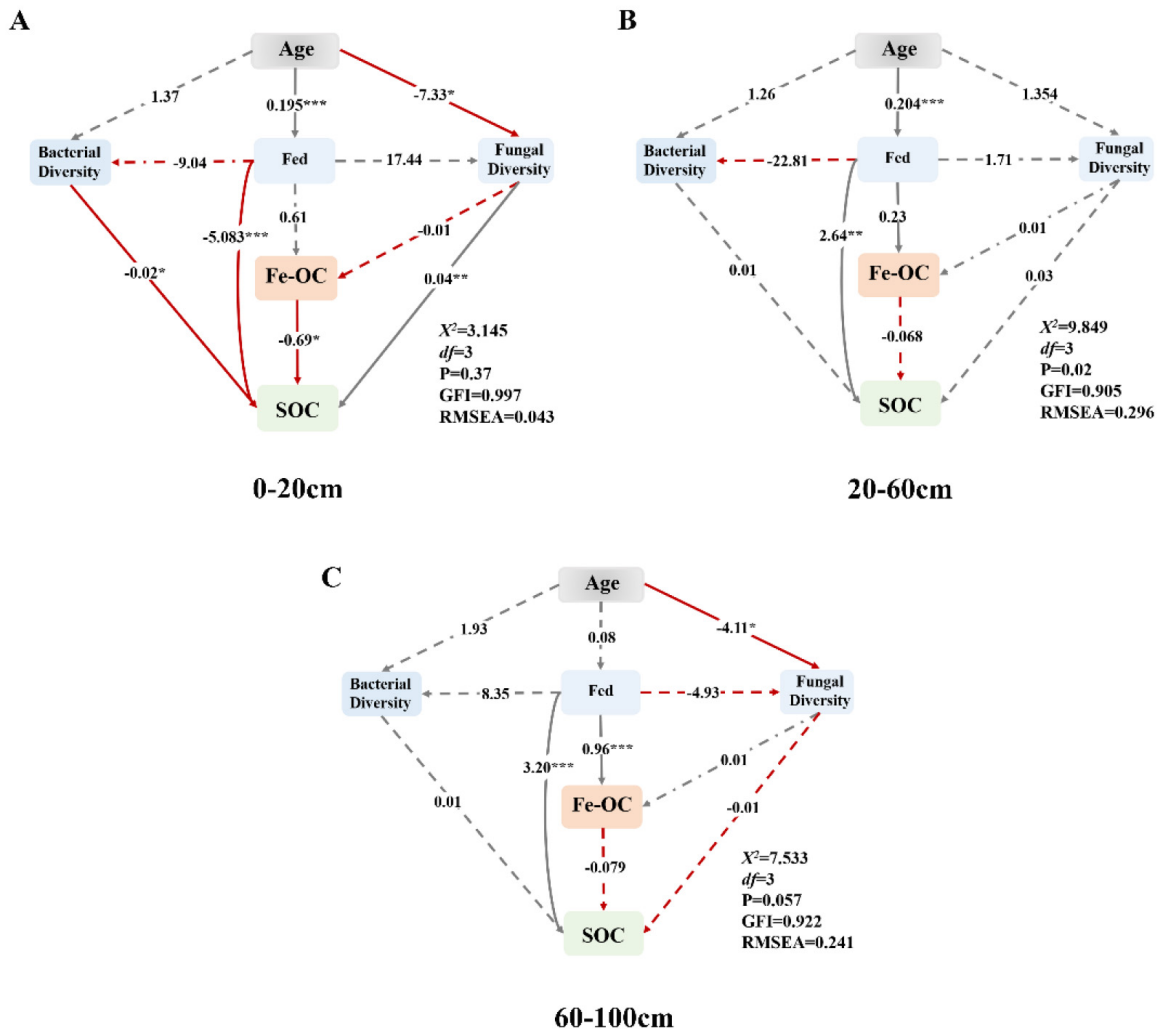
Structural equation modeling (SEM) for exploring the effects of Fe oxides and microorganisms in different soil layers [**(A)** 0–20 cm; **(B)** 20–60 cm; **(C)** 60–100 cm] on SOC and ironbound SOC. The red arrow indicates negative effects, and the black arrow indicates positive effects; values along the arrows are path coefficients. Solid lines represent significant correlations (*p* < 0.05), and dashed lines represent nonsignificant correlations (*p* > 0.05). ^*^, ^**^ and ^***^ indicate statistical significance at *p* < 0.05, *p* < 0.01 and *p* < 0.001, respectively.

Structural equation modeling (SEM) results (Figure 7) showed that stand age positively affected Fed content across all layers (β = 0.68, *p* < 0.001), and Fed positively directly affected Fe-OC (β = 0.72, *p* < 0.001). Conversely, Fe-OC was negatively correlated with SOC (*p* < 0.05), suggesting that increasing Fe-associated C may coincide with depletion of bulk SOC pools. In surface soils, bacterial diversity negatively impacted SOC (*p* < 0.05), with weaker positive effects in deeper layers. Fungal diversity positively affected SOC in surface and subsurface soils (*p* < 0.01) but negatively in deep layers, likely due to distinct ecological functions and environmental heterogeneity. The structural equation model revealed a depth-dependent pattern in the mechanistic pathways. While the models for the subsurface and subsoil layers did not achieve adequate fit, the surface-layer model demonstrated satisfactory fit, effectively illustrating the pathway by which stand age influences Fe-OC and bulk SOC through the modulation of iron oxide components.

## 4 Discussion

### 4.1 Forest control of SOC and Fe oxides

In this study, soil organic carbon (SOC) and its component contents were the highest in topsoil and decreased with increasing depth (Figure 1), which is consistent with the pattern that SOC is primarily supplied by surface litter input, while subsoil carbon relies mainly on root exudates and leaching (Rumpel and Kögel-Knabner, 2011). With increasing stand age, SOC peaked in 20-year-old stands, followed by 10-year-old stands, and was the lowest in 40-year-old stands (Figure 1A). This pattern is related to the peak aboveground litter and belowground carbon inputs in 20-year-old plantations (Dang et al., 2017), and may also be attributed to the saturation of adsorption capacity of microaggregates formed by the complexation of mineral-associated organic carbon (MAOC) with clay particles and iron oxides after 20–30 years (Sun et al., 2023). Accordingly, MAOC in this study also reached its maximum at age 20 (Figure 1F). The decline in SOC in 40-year-old stands may be further associated with enhanced priming effects, accelerated fine root turnover, and increased labile carbon release, as reflected by the highest dissolved organic carbon (DOC) concentration (Beidler et al., 2023; Cotrufo et al., 2015). Overall, SOC and its component contents followed the order: coniferous forest > mixed forest > broadleaved forest. Although broadleaved forests produce greater litter input and higher iron-bound carbon contents, their litter is of high quality (low C/N ratio, low lignin), leading to rapid decomposition and low net carbon sequestration. In contrast, coniferous litter is recalcitrant (high C/N ratio, rich in lignin and tannins), decomposes slowly, and favors higher SOC sequestration (De Feudis et al., 2025; Li et al., 2024). In contrast, the lower C/N ratio in broadleaved litter accelerates decomposition (Hüblová and Frouz, 2021). Notably, the higher iron-bound carbon content in broad-leaved forest soils suggests that Fe–carbon association alone does not guarantee long-term SOC stabilization; it may be that the Fe-bound carbon in broad-leaved forests is more labile or that rapid decomposition of non-Fe-bound carbon overshadows the protective effect (Han et al., 2024). Therefore, the dominant mechanism for SOC accumulation in coniferous forests appears to be litter recalcitrance rather than mineral protection by iron oxides in this study.

Fe oxides play a crucial role in stabilizing SOC through adsorption and coprecipitation mechanisms, forming organomineral complexes that reduce the bioavailability of SOC and favors its accumulation. In contrast, Fe oxides can also mediate SOC mineralization through dissimilatory Fe(III) reduction, which directly couples the decomposition of SOC to Fe redox cycling, releases Fe-bound SOC into soil solution, and increases its microbial availability. Therefore, Fe oxides exert a dual impact on SOC dynamics, either promoting its stabilization or inducig its loss through microbially driven mineralization (Wiesmeier et al., 2019). In our study, C:Fe molar ratios associated with Fe-bound SOC were >6 for most stand ages and forests, with only a few values <1 in 40-year-old broad-leaved forests (OBF; Figure 2I). With increasing soil depth, oxygen content decreases and pH shifts, favoring enhanced Fe(III) reduction. This may induce the dissolution of iron oxides and the release of previously protected carbon, leaving mostly crystalline iron oxides with low affinity for dissolved organic carbon (DOC). Consequently, the content of iron-bound carbon declines in deep soils, accompanied by a reduced C:Fe ratio (Figure 2F). Coniferous litter is characterized by high acidity, a high C/N ratio, and rich phenolic compounds, which favor the occurrence of poorly crystalline iron phases (Figure 2I). These iron phases form stable inner-sphere complexes with DOC, leading to high carbon stability. In contrast, broadleaf forests produce a large quantity of litter and maintain relatively high soil pH, which facilitates the formation of crystalline iron oxides. Diverse DOC molecules can compete for sorption sites; although iron-bound carbon content is high, the C:Fe ratio is relatively low, and carbon is mainly bound via outer-sphere complexation, resulting in weaker carbon stability (Luo et al., 2024). During coprecipitation, Fe oxides interact with DOC via coordination bonds, hydrogen bonding and physical encapsulation, which effectively slow SOC decomposition (Schulin et al., 2007). Furthermore, the geochemical structure of SOC may undergo hydrogenation and polymerization, which further enhances its resistance to microbial decomposition (Li et al., 2023).

To further clarify the underlying mechanism, nuclear magnetic resonance (NMR) spectroscopy studies have shown that the binding strength in coprecipitation is mainly controlled gaveled by carboxyl functional groups (Henneberry et al., 2012). Plant litter rich in lignin inputs increases the carboxyl content and aromaticity of SOC in top soils, thereby enhancing the affinity between SOC and Fe oxides via multidentate ligand exchange (Wan et al., 2019). This multi-carboxyl exchange not only improves SOC sequestration efficiency but also explains the higher Fe oxide content observed in surface soils. Consequently, the stabilizing effect of Fe oxides on SOC is most pronounced in topsoils, where reactive Fe and carboxyl-rich organic matter co-occur. This process substantially increases SOC's resistance to desorption and oxidation, allowing it to form mono- or multi-layer adsorption structures on Fe oxide surfaces, thus ensuring its long-term stability in soils and sediments (Curti et al., 2021).

### 4.2 The relationship between microbial communities and SOC stability

The structure and functional diversity of soil microbial communities have a significant impact on the formation and stability of SOC. Microorganisms respond to environmental changes through various life strategies, thereby regulating the SOC dynamic (Delgado-Baquerizo et al., 2016). We showed that the richness of bacteria and fungi both exhibited a decreasing trend with the increase in soil depth (Figure 4), consistent with reduced organic substrates and biological activity in deeper horizons. Across stand-age stages, microbial diversity was higher in 10-year-old and 20-year-old forests than in 40-year-old forests, likely reflecting more favorable edaphic conditions (e.g., soil pH and root distribution) as well as higher quality and quantity of nutrient inputs (e.g., labile organic matter from litter and root exudates) in the younger stands, which enhance microbial metabolic activity and diversity. Soil acidification reduces microbial metabolic activity, increases physiological stress, and ultimately selects for a smaller pool of stress-tolerant species, thereby decreasing microbial diversity (Zhou et al., 2021). In addition, changes in microbial communities may lag behind shifts in vegetation and soil properties, with dominant tree species exerting strong control over microbial community composition (Urbanová et al., 2015). Therefore, there was no significant change in the richness of soil microorganisms across different stand ages in this study.

From a life-history perspective, soil microorganisms can be classified into r-strategists (copiotrophs, fast-growing) and K-strategists (oligotrophs, slow-growing), based on their mineralization capacity and growth rate (Li H. et al., 2021). K-strategists (e.g., *Acidobacteria, Actinobacteria and Chloroflexi*) are well adapted to nutrient-poor soils with low SOC mineralization rates and efficiently utilize recalcitrant carbon with higher substrate-use efficiency (Shao et al., 2021). We showed that the high abundance of K-strategist coincided with lower bacterial diversity (Figures 3A–C), yet these soils often have relatively high soil organic carbon (SOC) content. This phenomenon can be attributed to a stabilizing effect of K-strategist bacteria on SOC stability, whereby slower growth and more efficient resource use reduce the SOC mineralization rate (Osburn et al., 2021). The absence of a similar pattern in fungal communities may reflect the strong competitiveness and adaptability of certain fungal guilds (e.g., ectomycorrhizal fungi) in oligotrophic and acidic environments (Maillard et al., 2023), which can maintain high functional diversity even under resource limitation. *Firmicutes* and *Methylomirabilota* showed opposite trends with increasing soil depth (Supplementary Figure S6). Under anoxic conditions, Firmicutes can perform anaerobic fermentation to produce gases such as methane, potentially influencing SOC stability by enhancing carbon loss through gaseous emissions (Kajla et al., 2022). In contrast, *Methylomirabilota* can use CH_4_ as a carbon and energy source, converting CH_4_ into CO_2_ and SOC through anaerobic methane oxidation (Ettwig et al., 2010). This process not only mitigates CH_4_ emission, but also converts CH_4_ into SOC, directly contributing to soil carbon sequestration (Lee et al., 2020).

Microbial functional potential is increasingly recognized as a key indicator of ecosystem multifunctionality, underpinning the provision of ecosystem services (Philippot et al., 2021). Functional prediction by PICRUSt2 revealed that the relative abundances of functional genes associated with nitrogen fixation, amino acid metabolism, lipid metabolism, and carbohydrate metabolism were relatively high in mixed forests. These metabolic pathways are central to microbial primary metabolism and soil nutrient cycling, implying that mixed forests may support microbial communities with diverse functions and high biogeochemical potential. Functional redundancy is crucial for buffering ecosystems against disturbance and maintaining functional stability (Lu et al., 2025). Mixed forests with high functional redundancy can theoretically maintain the long-term stability of soil organic carbon (SOC) by distributing carbon and nitrogen transformation pathways across multiple metabolic routes, thereby avoiding over-reliance on a single transformation pathway. In contrast, monoculture plantations with low functional redundancy are prone to carbon loss due to metabolic bias (Ding et al., 2024; Shi et al., 2025). For example, the predicted aromatic compound degradation pathways accounted for a relatively high proportion in coniferous forest samples (Figure 5A), accompanied by a high abundance of cellulase-related genes. This may coincide with the sharp decline of labile soil organic carbon (e.g., POC and ROC) after reaching a peak at 10 years of stand age. In the deep soil of 40-year-old broad-leaved forest (OBF, Figure 5D), the predicted abundances of metabolic pathways including chlorophyll synthesis (PWY-5531) and lipid metabolism (PWY-7007) were relatively high. We speculate that these pathways may compensate for the loss of diversity and help maintain the high soil organic carbon content (67.4 g·kg^−1^) in deep soil. Overall, our results indicate that monoculture plantations tend to support relatively narrow metabolic functions, whereas mixed forests may exert positive effects on long-term carbon sequestration via microbial metabolic complementarity and diverse plant-derived carbon sources (Xu et al., 2024).

### 4.3 Contribution of Fe oxide-microbial interactions to SOC stability

Iron-SOC complexation significantly shapes the structure and function of microbial communities by regulating the bioavailability of SOC, microenvironmental conditions, and redox status (Han et al., 2024). Notably, although structural equation modeling indicated that Fed significantly promoted the formation of Fe-bound organic carbon, our correlation analysis revealed a negative relationship between Fe-OC and total soil organic carbon (SOC). One plausible explanation for this seemingly contradictory result is the dilution effect within the soil carbon pool: as total soil organic carbon (SOC) accumulates, the accumulation rate of non-Fe-bound organic carbon fractions (e.g., particulate organic carbon, POC) may outpace that of Fe-bound fractions, thereby reducing the relative contribution of Fe-OC to the total carbon pool (Dungait et al., 2012; Zhou et al., 2023). Notably, the space-for-time substitution employed in this study does not allow direct quantification of the accumulation rates of individual carbon fractions; thus, this dilution hypothesis remains to be further validated by long-term *in situ* observations or isotopic tracing in future research. Nevertheless, our findings clearly demonstrate that although iron oxides effectively complex and protect specific organic carbon fractions, the overall size of the total soil carbon pool is primarily regulated by the dynamics of other carbon components. *Acidibacter*, as an acidophilic and obligate heterotrophic bacterium, is capable of reducing ferric iron [Fe(III)] (Wu et al., 2018). Our results showed that acidophilic bacterial genera such as *Acidobacteriota* and *Acidothermus* were significantly negatively correlated with Fed, while SOC was significantly and positively correlated with *Acidothermus*, which manifested as a trend for sites with high contents of acidophilic bacteria and SOC to have low Fed content and reduced bacterial richness (Figure 6C, Supplementary Figure S8). This negative correlation suggests that acidophilic bacteria may weaken the physical protection of iron to a certain extent by reducing and dissolving iron oxides, thereby increasing the bioavailability of organic carbon. *Acidothermus* decomposes complex organic matter such as cellulose to release DOC, which binds to Fe oxides and forms stable organo-mineral complexes, thereby promoting the stability and accumulation of SOC (Kleber et al., 2021). Once DOC is protected by the complexes, however, its availability for microbial metabolism declines, thereby inhibiting the SOC priming effect (Adhikari et al., 2019). The limited accessibility of DOC favors K-strategists including most fungi, which can acquire carbon and nutrients via extensive hyphae networks (Yu et al., 2018).

Soils rich in Fe oxides are dominated by fast-growing microorganisms such as *Proteobacteria, Actinobacteria*, and *Mortierellomycota* (Converse et al., 2015). *Mortierella*, a major genus within *Mortierellomycota*, is often correlated with Bacillus because its hyphae can both decompose complex carbon sources and provide “highways” for bacterial dispersal (Simon et al., 2017). In addition, both Proteobacteria and Actinobacteria were positively correlated with SOC (Figure 6A) and negatively correlated with Fe oxides, while *Mortierellomycota* was negatively correlated with Fe oxides (Figure 6B). These correlation patterns indicate that complex feedback relationships may exist between microbial communities and iron oxides. Environments with high iron content select for specific microbial taxa, whose metabolic activities in turn may alter the speciation of iron via complexation, reductive dissolution, or both. Therefore, SOC accumulation in soil with high Fe oxide content may impose stronger demands on the carbon degradation capacity of microorganisms, driven by the limited availability of carbon resources. Such environmental conditions may be more favorable for the survival and reproduction of K-strategist bacteria, which can effectively utilize low-concentration carbon sources and maintain a competitive advantage in resource-limited environments.

SOC fractions, Fe oxide contents, and microbial communities of the three plantations in the red soil region exhibited significant differences across stand ages and soil layers (Figures 1, 2). The interactions among these factors may underlie their unique carbon sequestration mechanisms. Soil moisture, biological oxygen demand, and dissolved organic carbon released from litter rich in acidic groups collectively regulate the transformation of iron oxides (Perez-Fodich and Derry, 2019). Coniferous forest litter is rich in lignin, tannins, and waxes, with a high carbon-to-nitrogen ratio (C/N) and strong resistance to decomposition. This may drive microorganisms to preferentially utilize available nitrogen sources for carbon decomposition (Zhang J. et al., 2024), thereby achieving coupled carbon and nitrogen sequestration and stabilizing soil organic carbon through the physical protection mechanism of soil aggregates (Li C. et al., 2024). High lignified carbon input, acidic soil conditions, and constrained nutrient mineralization collectively create oligotrophic environments, which may significantly enhance the stability of the soil organic carbon pool. However, iron oxides play a relatively limited role in carbon sequestration in coniferous forests, which is consistent with the observation that both iron oxide content and iron-bound soil organic carbon content are lower compared with mixed and broad-leaved forests (Figure 2). This may be due to the lower organic matter content in coniferous forests, the more recalcitrant nature of coniferous litter compared to broad-leaved litter, and its fewer active functional groups capable of interacting with soil minerals (Kaiser et al., 2011). In contrast, broad-leaved and mixed forests have high contents of cellulose and hemicellulose, which are readily decomposed into small-molecule substrates such as organic acids for rapid microbial utilization (Chen et al., 2024).

Metabolic analysis predicted that significant mineral-associated soil organic carbon (SOC) stabilization mechanisms may occur in the deep soil of broad-leaved forests, whereas surface SOC is mineralized to CO_2_ via microbial degradation and metabolic processes (Chai et al., 2024). This vertical difference is presumed to originate from its well-developed root system. Rhizosphere microbes in deep soil synthesize carbon and nitrogen nutrients via the betaine degradation pathway (PWY-3661) to cope with drought stress (Figure 5C). Our functional prediction also revealed active nitrification-related genes in broad-leaved forest soils (Figures 5B, C), implying that the deep soil still retains considerable carbon sequestration potential, which is consistent with other findings on carbon sequestration in broad-leaved forests (Zhu et al., 2024). Mixed forests integrate the advantages of monoculture plantations: the surface layer benefits from heterogeneous litter input, while the deep layer stabilizes SOC through iron oxide protection. Microbial metabolic pathways predicted in mixed forests are mainly involved in nucleotide and amino acid synthesis, which may not only meet the nutritional requirements of microbial communities but also promote nutrient mineralization and plant uptake. Collectively, coniferous forests may stabilize SOC primarily through the input of chemically recalcitrant carbon and the maintenance of oligotrophic conditions; in contrast, mixed and broad-leaved forests may sequester SOC via complex microbial metabolism and mineral protection. Based on our findings, mixed-species plantations are recommended for long-term soil carbon sequestration in subtropical red soil regions, as they combine relatively high SOC storage with enhanced Fe-OC coprecipitation and mineral protection, unlike coniferous forests which rely mainly on litter recalcitrance. SOC peaked at 20 years and declined by 40 years, suggesting an optimal rotation age of approximately 20 years for maximizing surface carbon stocks. However, deep soil layers (60–100 cm) in 40-year-old mixed and broad-leaved forests maintained high SOC and MAOC, representing a substantial and stable carbon sink that should not be overlooked.

## 5 Conclusions

This study provides evidence for interactions between Fe oxides and microbial communities in SOC accumulation and stabilization in forests of red soil regions. Our main findings are: (1) SOC sequestration potential exhibits a non-linear pattern with stand age, peaking at 20 years; (2) forest type is associated with distinct stabilization pathways: coniferous forests show patterns consistent with litter quality and oligotrophic conditions playing a role, broadleaf forests exhibit links between enzymatically mediated Fe-OC coprecipitation (via UMO) and deep-root metabolism, while mixed forests display synergistic coupling between acidophilic bacteria and Fe oxides; (3) microbial communities shift from r- to K-strategists with increasing stand age, a trend associated with greater carbon storage; and (4) deep soil layers, especially in mature mixed forests, represent a substantial carbon sink that warrants further attention.

These findings suggest that promoting mixed-species forests and extending rotation periods beyond 20 years may contribute to SOC stabilization in red soil regions. Furthermore, our results highlight the importance of considering mineral-organic-microbial interactions for accurately predicting soil carbon dynamics under climate change. This work thus provides a empirical basisfor designing strategies to manage plantation forests as nature-based solutions for climate change mitigation.

## Data Availability

The 16S rRNA gene and ITS sequences were deposited in the National Center for Biotechnology Information (NCBI) under BioProject accession number PRJCA043171.
