# Supplementary material for: Forest type and stand age co-regulate iron-associated carbon and microbial life-history strategies in red soils
Source: Front Microbiol. 2026 Apr 30;17:1780540. doi: 10.3389/fmicb.2026.1780540 (PMC13171523; doi:10.3389/fmicb.2026.1780540)
Supplement: Supplementary file 1 [file Data_Sheet_1.docx]

**Forest type and stand age co-regulate iron-associated carbon and microbial life-history strategies in red soils**

Fuxing Tan, Renlu Liu,Yian Wang, ZhiJun Cao,Houwen Zhang, Li Yin,Genhe He*

Key Laboratory of Jiangxi Province for Functional Biology and Pollution Control in Red Soil Regions, School of Life Sciences, Jinggangshan University, Ji'an 343009, China

# **Figures:**


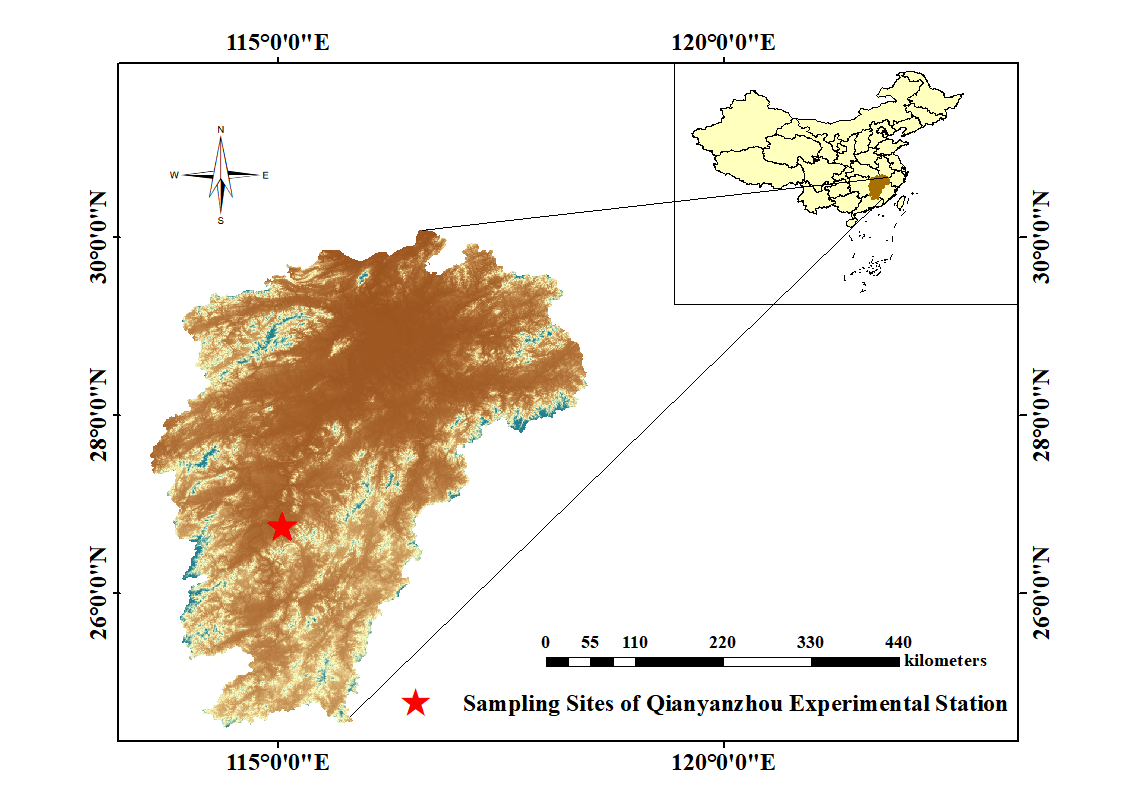


**Fig.S1 The Location of Soil Sampling Points at Qianyanzhou Experimental Station**


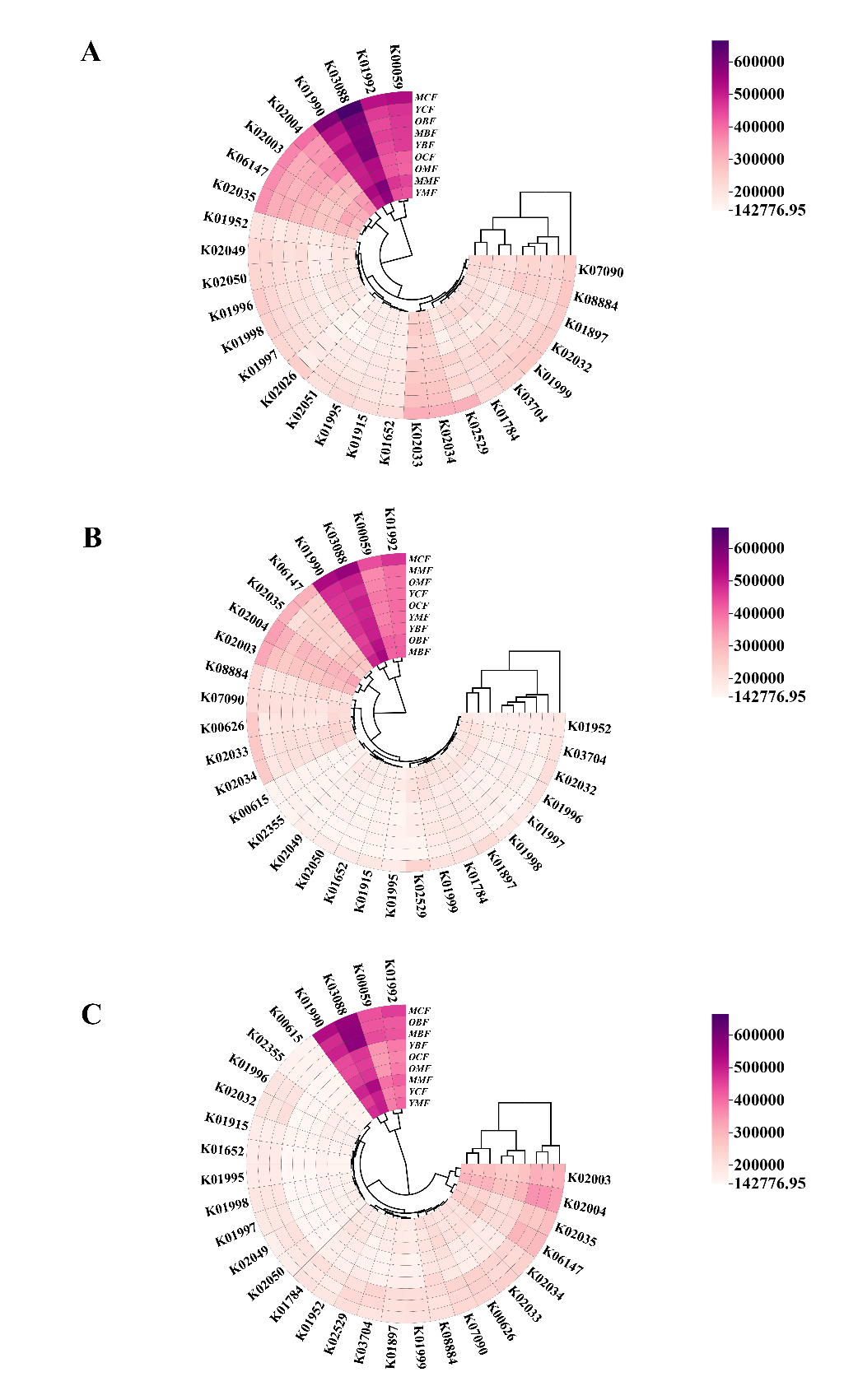


**Fig.S2 Abundance distributions of bacterial KEGG Orthology (KO) functional genes (A-C) at different forest ages and in different soil layers predicted by PICRUSt2.A:0-20cm; B:20-60cm; C:60-100cm. OCF, 40 year coniferous forest; MCF, 20 year coniferous forest; YCF, 10 year coniferous forest; OMF, 40 year mixed forest; MMF, 20 year mixed forest; YMF, 10 year mixed forest; OBF, 40 year broad-leaved forest; MBF, 20 year broad-leaved forest; YBF, 10 year broad-leaved forest.**

**
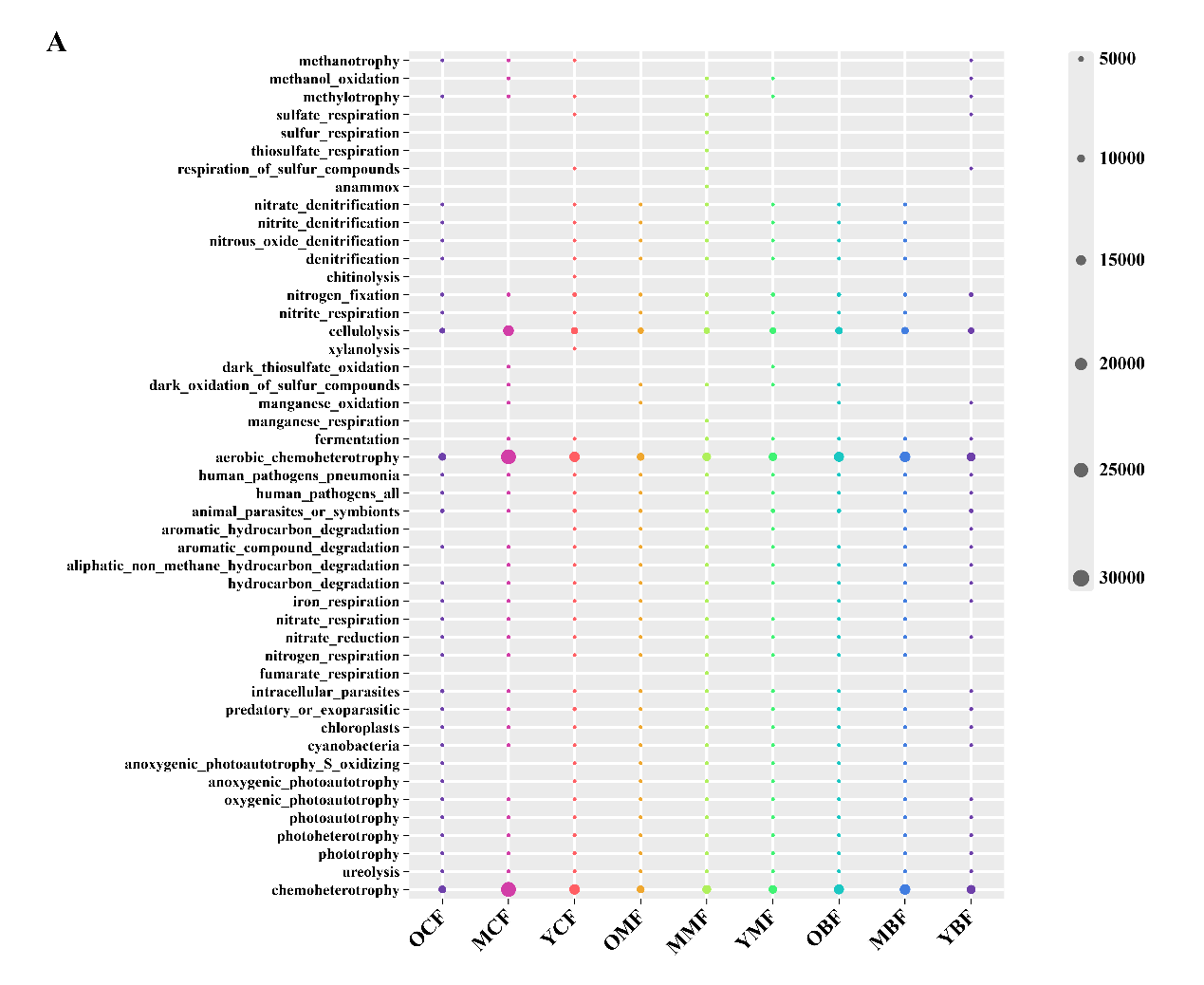
**

**
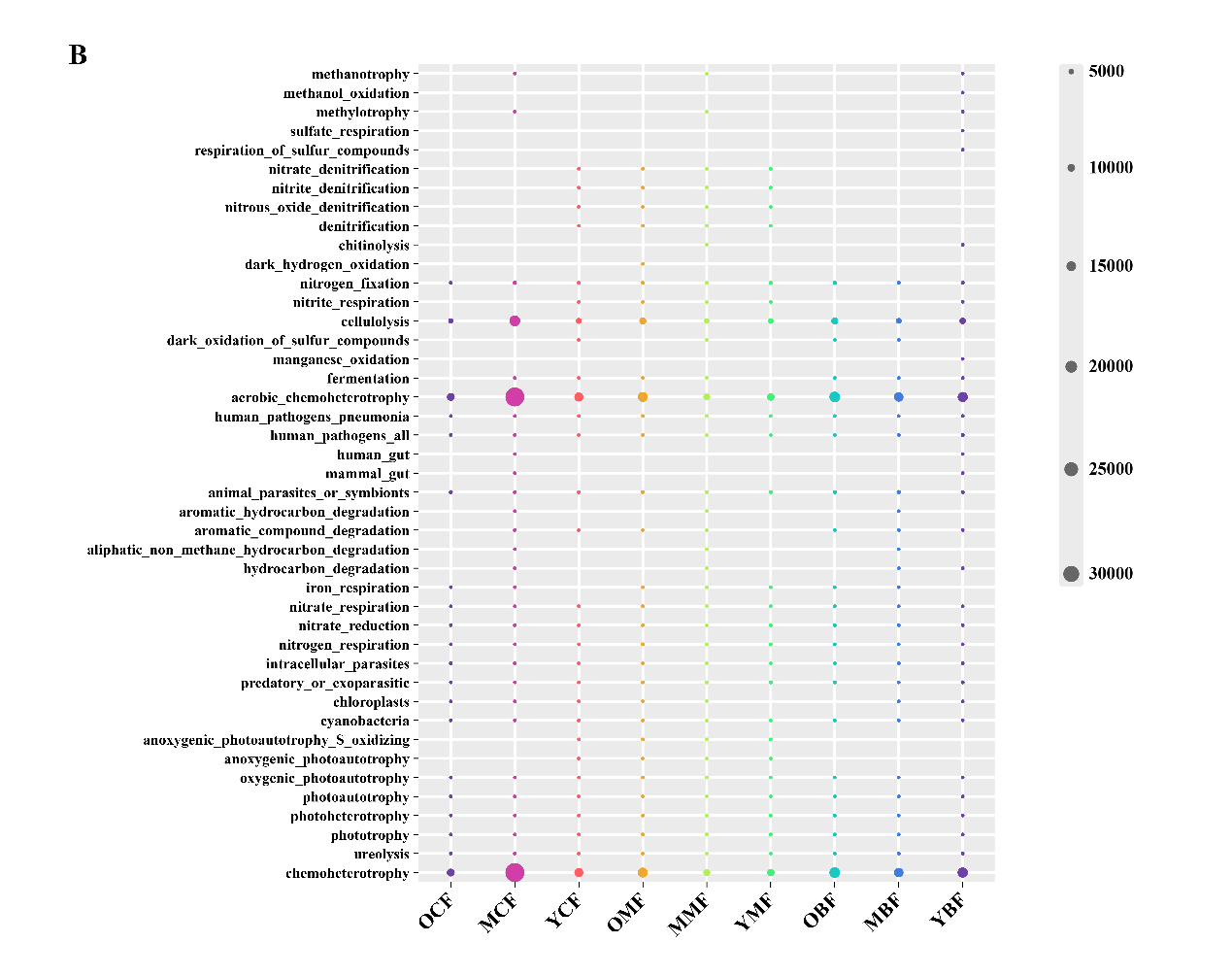
**

**
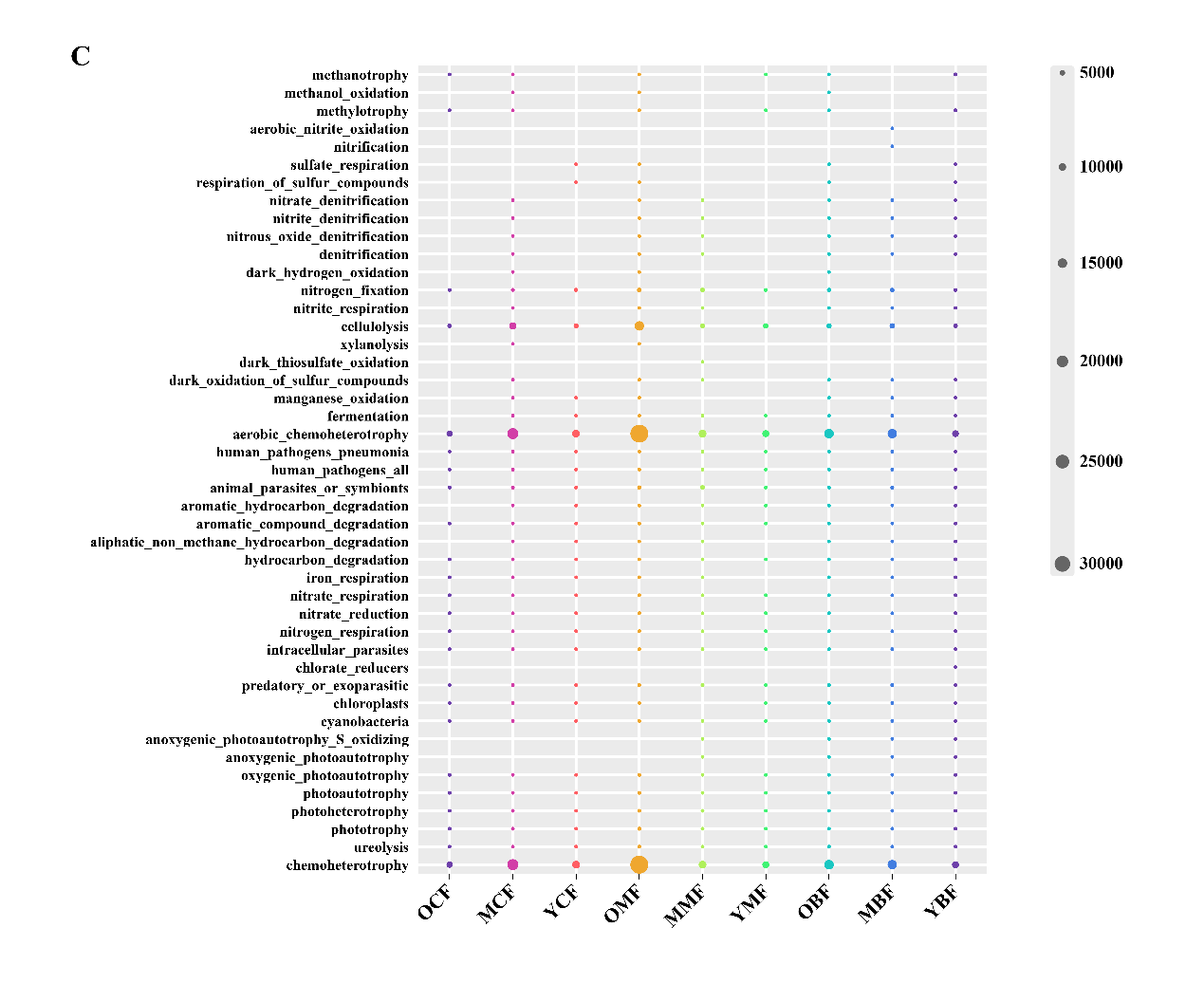
**

**Fig.S3 Functional potentials of soil bacterial communities under different forest ages and soil layer depths:(A-C) Relative abundances of ecological functional groups predicted by FAPROTAX. A:0-20cm; B:20-60cm; C:60-100cm. OCF, 40 year coniferous forest; MCF, 20 year coniferous forest; YCF, 10 year coniferous forest; OMF, 40 year mixed forest; MMF, 20 year mixed forest; YMF, 10 year mixed forest; OBF, 40 year broad-leaved forest; MBF, 20 year broad-leaved forest; YBF, 10 year broad-leaved forest.**

**
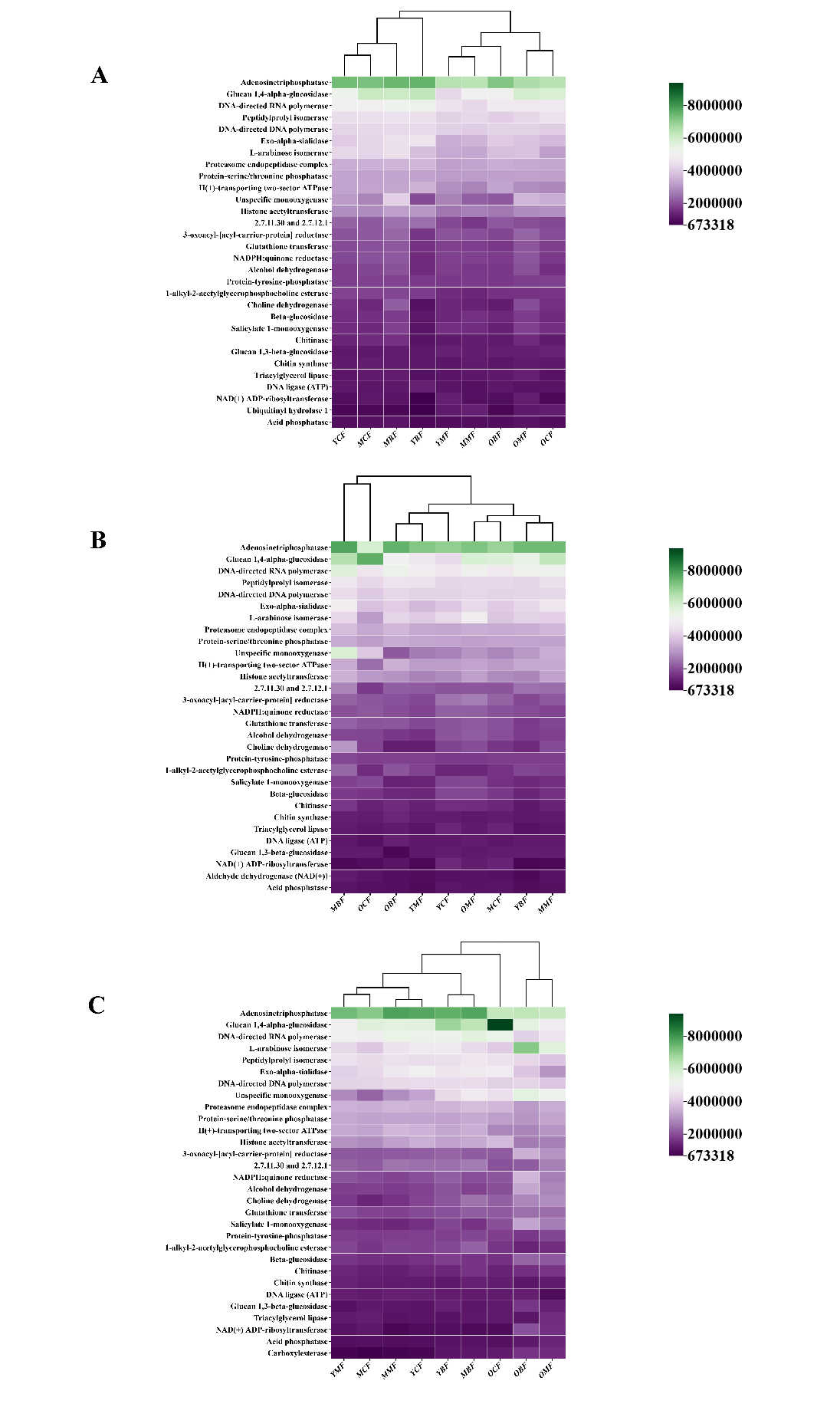
**

**Fig.S4 Abundance distributions of fungal enzyme (A-C) abundances at different forest ages and in different soil layers predicted by PICRUSt2.A:0-20cm; B:20-60cm; C:60-100cm. OCF, 40 year coniferous forest; MCF, 20 year coniferous forest; YCF, 10 year coniferous forest; OMF, 40 year mixed forest; MMF, 20 year mixed forest; YMF, 10 year mixed forest; OBF, 40 year broad-leaved forest; MBF, 20 year broad-leaved forest; YBF, 10 year broad-leaved forest.**


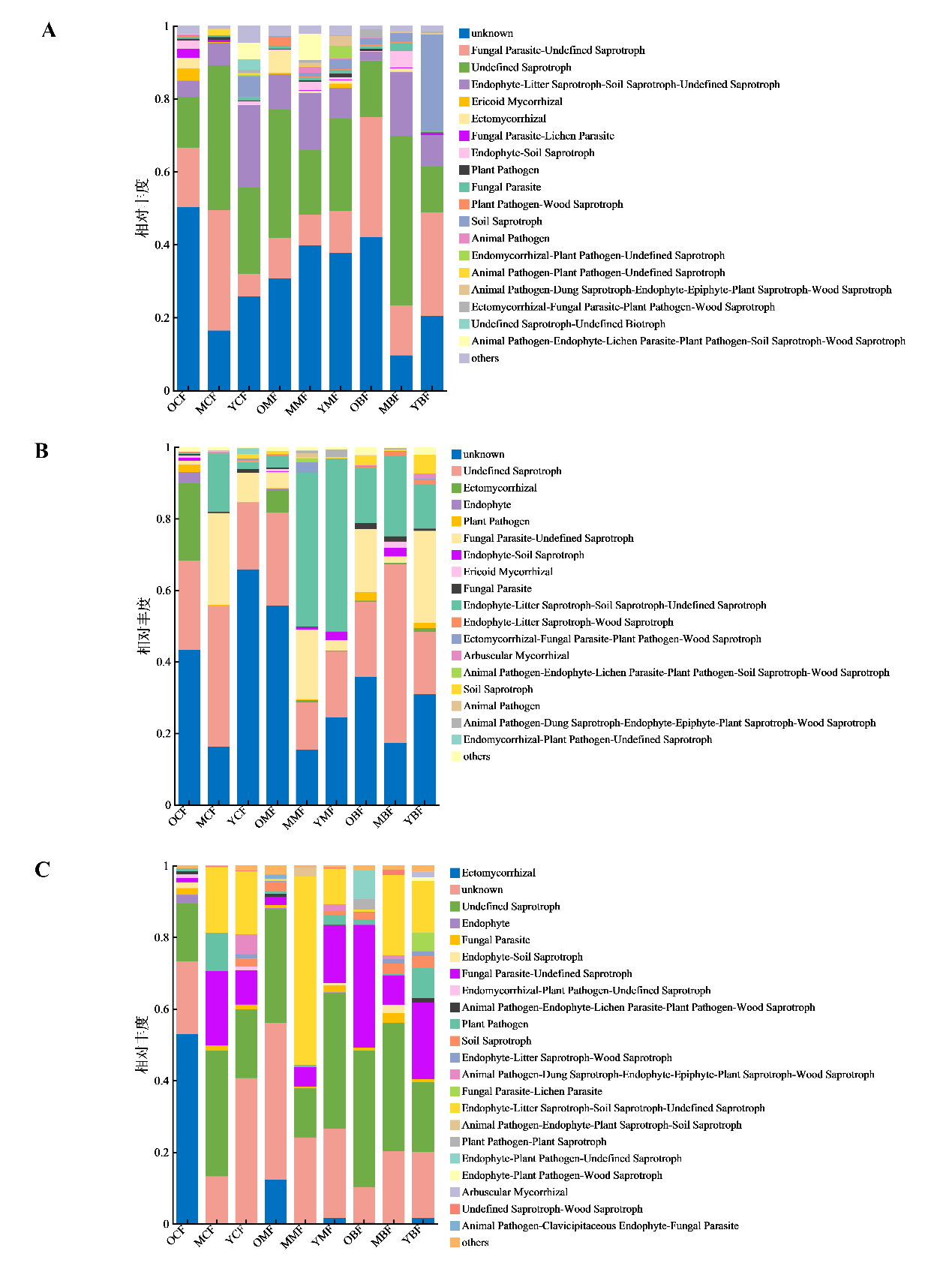


**Fig. S5 Relative abundances of ecological functional genes of soil fungi predicted by FUNGuild under different forest ages and soil layer depths.A 0-20cm; B20-60cm; C60-100cm.OCF, 40 year coniferous forest; MCF, 20 year coniferous forest; YCF, 10 year coniferous forest; OMF, 40 year mixed forest; MMF, 20 year mixed forest; YMF, 10 year mixed forest; OBF, 40 year broad-leaved forest; MBF, 20 year broad-leaved forest; YBF, 10 year broad-leaved forest.**

**
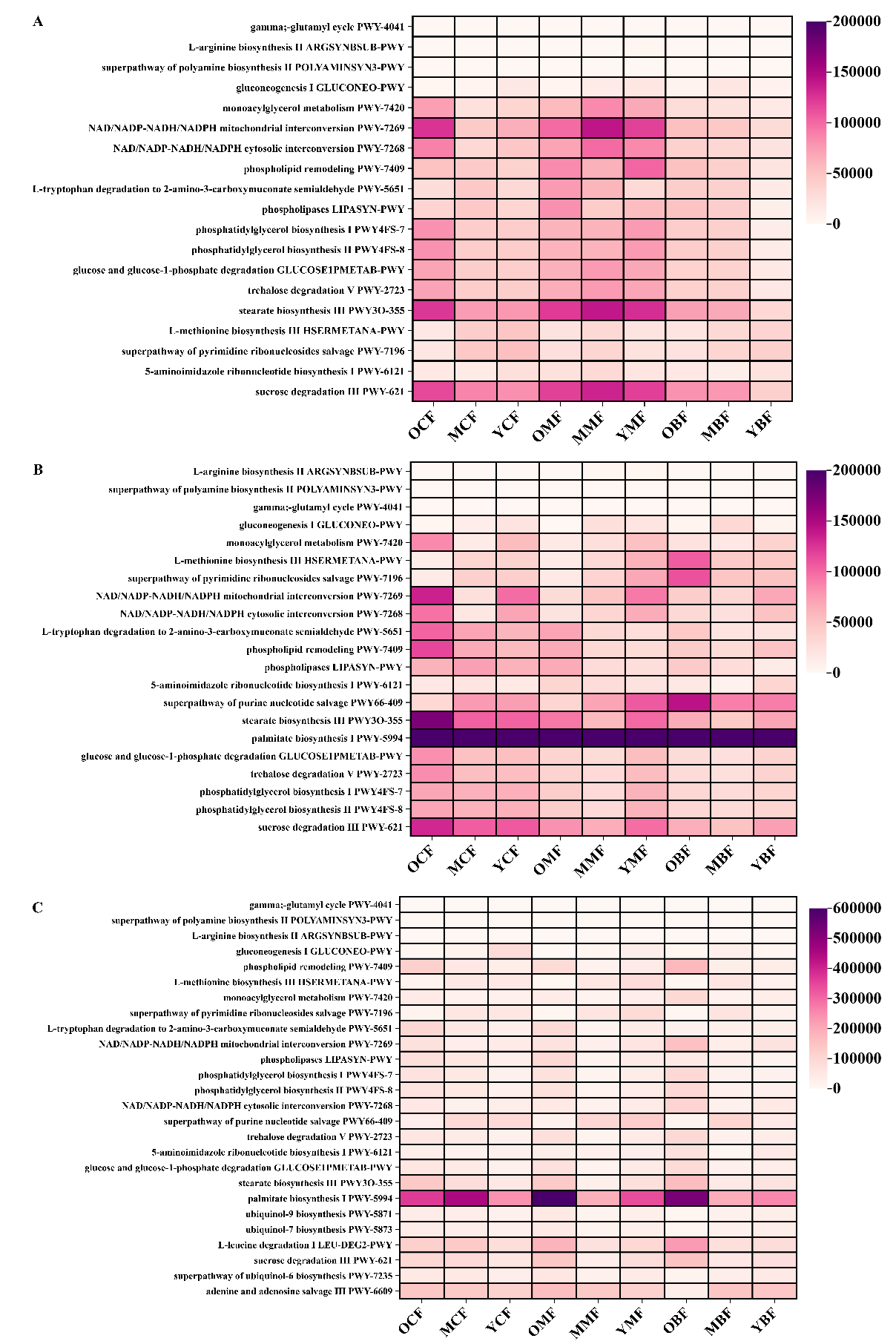
**

**Fig. S6 Functional potentials of soil fungal communities under different forest ages and soil layer depths:(A-C) Relative abundances of metabolic pathways predicted by PICRUSt2 based on the MetaCyc database. A:0-20cm; B:20-60cm; C:60-100cm. OCF, 40 year coniferous forest; MCF, 20 year coniferous forest; YCF, 10 year coniferous forest; OMF, 40 year mixed forest; MMF, 20 year mixed forest; YMF, 10 year mixed forest; OBF, 40 year broad-leaved forest; MBF, 20 year broad-leaved forest; YBF, 10 year broad-leaved forest.**

**
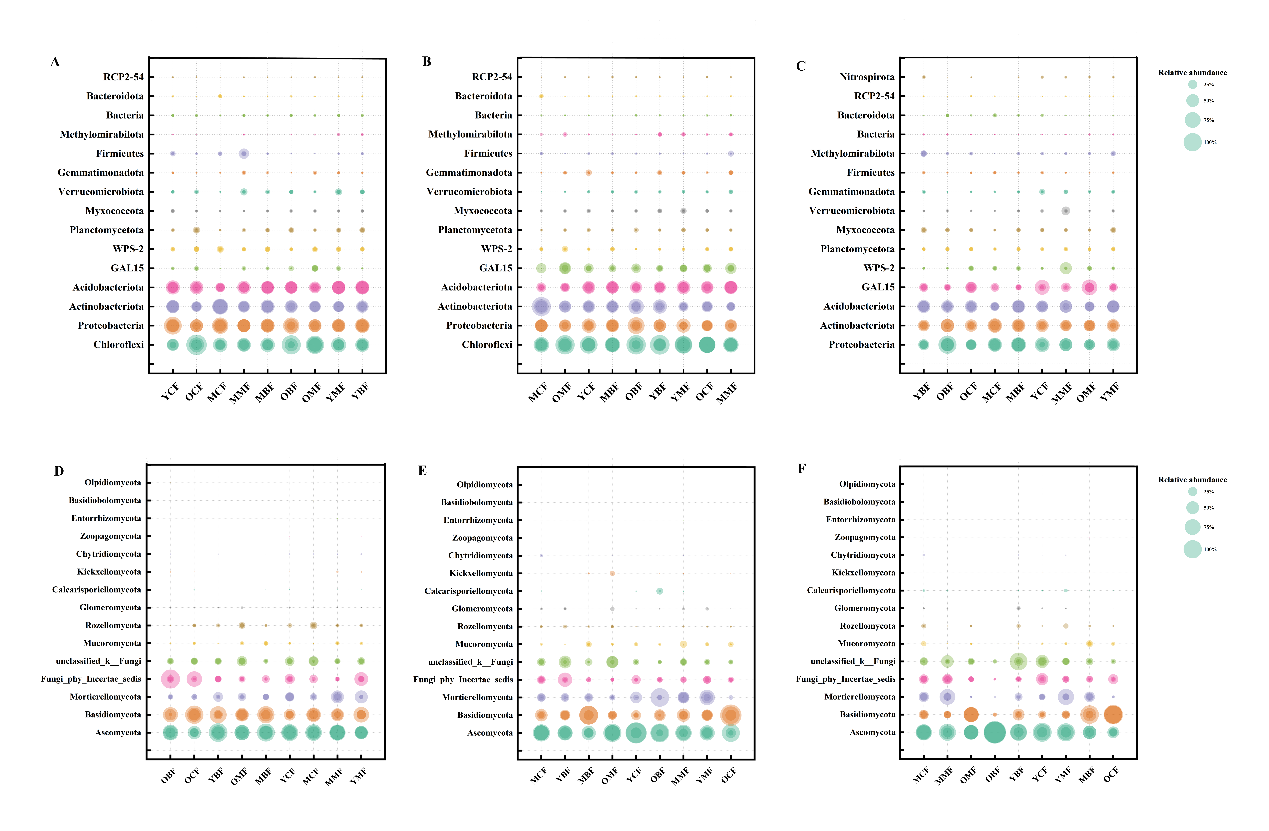
**

**Fig. S7Bubble plot showing the relative abundance of dominant phylum-level bacteria (A-C) and fungi (D-F) across different soil layers in plantation forests of varying stand ages .A,D 0-20cm; B,E 20-60cm; C,F 60-100cm. OCF, 40 year coniferous forest; MCF, 20 year coniferous forest; YCF, 10 year coniferous forest; OMF, 40 year mixed forest; MMF, 20 year mixed forest; YMF, 10 year mixed forest; OBF, 40 year broad-leaved forest; MBF, 20 year broad-leaved forest; YBF, 10 year broad-leaved forest.**

**
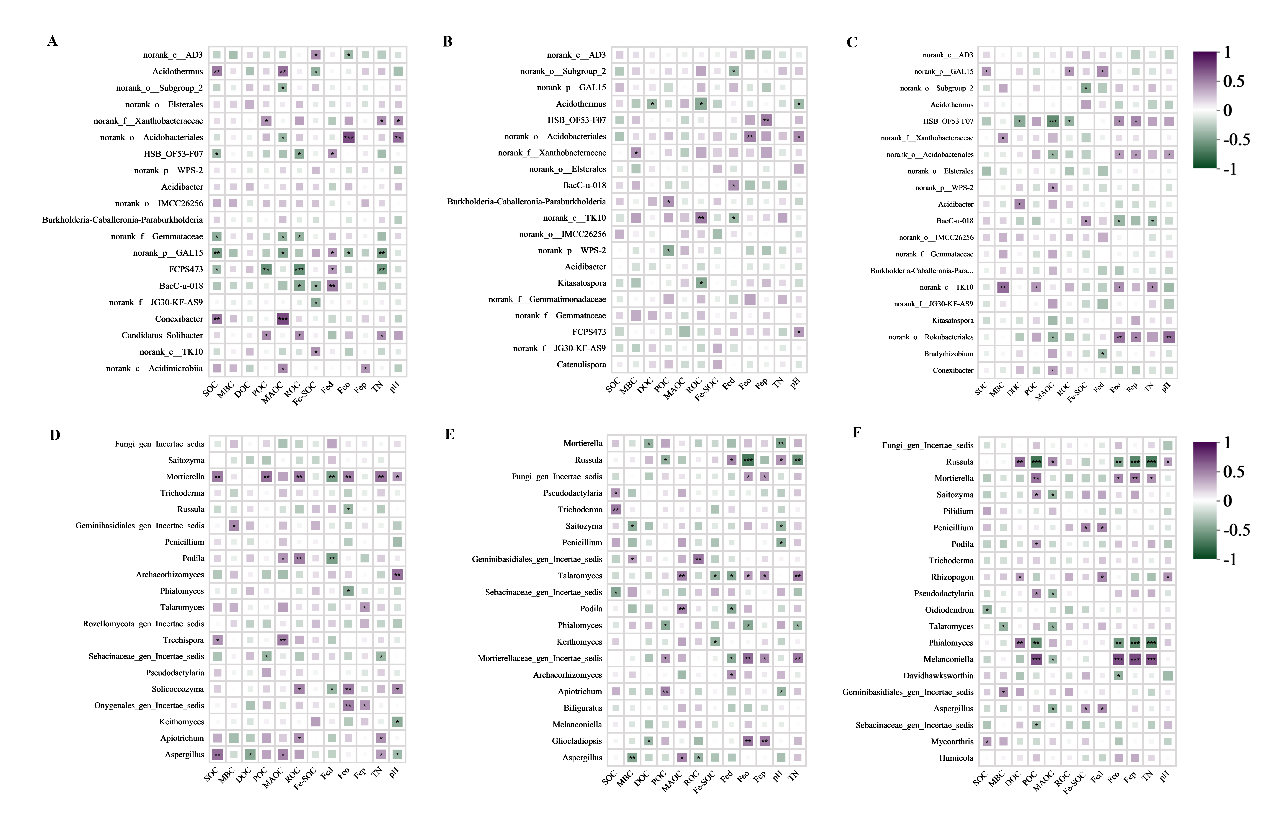
**

**Fig. S8Analysis of the correlation between dominant genus-level bacteria (A-C) and fungi (D-F) and environmental factors in different soil layers. A,D 0-20cm; B,E 20-60cm; C,F 60-100cm. OCF, 40 year coniferous forest; MCF, 20 year coniferous forest; YCF, 10 year coniferous forest; OMF, 40 year mixed forest; MMF, 20 year mixed forest; YMF, 10 year mixed forest; OBF, 40 year broad-leaved forest; MBF, 20 year broad-leaved forest; YBF, 10 year broad-leaved forest.**

# **Table**

**Table. 1 Physical and chemical properties at different forest ages and soil depths.**

|  | Depth | SOC  (g·kg^-1^) | MBC  (mg·kg^-1^) | DOC  (mg·kg^-1^) | ROC  (g·kg^-1^) | POC  (g·kg^-1^) | MAOC  (g·kg^-1^) | TN  (g·kg^-1^) | pH | SWC |
| --- | --- | --- | --- | --- | --- | --- | --- | --- | --- | --- |
| OCF | 0-20cm | 33.51±1.77^b^ | 46.6±5.04^a^ | 18.07±2.85^a^ | 3.87±0.44^cd^ | 7.18±0.29^d^ | 26.33±1.53^bcde^ | 0.6±0.03^c^ | 4.49±0.05^bcd^ | 16±1%^cd^ |
|  | 20-60cm | 17.18±0.94^b^ | 38.25±5.43^a^ | 11.92±1.51^a^ | 13.28±1.03^cd^ | 8.57±0.87^d^ | 8.62±1.81^bcde^ | 0.4±0.02^c^ | 4.75±0.06^bcd^ | 16±1%^cd^ |
|  | 60-100cm | 17.68±1.35^b^ | 20.67±1.06^a^ | 10.64±0.34^a^ | 7.93±0.86^cd^ | 1.62±0.39^d^ | 16.06±1.74^bcde^ | 0.3±0.04^c^ | 4.86±0.18^bcd^ | 15±2%^cd^ |
| MCF | 0-20cm | 84.1±4.17a^b^ | 42.39±2.76^b^ | 9.57±0.67^d^ | 22.94±3.51b^cd^ | 23.86±3.87^bcd^ | 60.24±2.69^a^ | 1.46±0.36^ab^ | 4.31±0.02^e^ | 20±1%^bc^ |
|  | 20-60cm | 29.77±1.68^ab^ | 8.53±0.41^b^ | 6.77±0.2^d^ | 2.85±0.88^bcd^ | 9.83±0.97^bcd^ | 19.94±2.61^a^ | 0.79±0.23^ab^ | 4.28±0.01^e^ | 18±1%^bc^ |
|  | 60-100cm | 17.43±0.94^ab^ | 9.54±1.1^b^ | 6.73±0.69^d^ | 5.81±2.53^bcd^ | 5.66±0.72^bcd^ | 11.77±1.22^a^ | 0.38±0.03^ab^ | 4.58±0.04^e^ | 14±0%^bc^ |
| YCF | 0-20cm | 73.51±2.92^ab^ | 52.74±0.66^a^ | 15.61±2.28^ab^ | 33.05±0.99^ab^ | 28.41±1.04^ab^ | 45.1±3.92^abc^ | 1.73±0.08^a^ | 4.78±0.14^bcd^ | 17±1%^d^ |
|  | 20-60cm | 31.14±2.62^ab^ | 34.91±0.46^a^ | 12.23±2.81^ab^ | 12.43±0.62^ab^ | 18.81±2.3^ab^ | 12.33±3.48^abc^ | 1.02±0.03^a^ | 4.63±0.07^bcd^ | 14±0%^d^ |
|  | 60-100cm | 22.79±1.8^ab^ | 16.09±0.25^a^ | 10.07±0.88^ab^ | 14.45±1.45^ab^ | 10.09±0.19^ab^ | 12.71±1.68^abc^ | 0.78±0.05^a^ | 4.63±0.02^bcd^ | 13±0%^d^ |
| OMF | 0-20cm | 24.91±2.12^a^ | 15.44±0.85^b^ | 13.19±2.52^ab^ | 12.78±3.16^abcd^ | 14.76±0.55^cd^ | 10.15±3.9^cde^ | 0.62±0.06^c^ | 4.54±0.11^bc^ | 20±0%^b^ |
|  | 20-60cm | 69.89±1.88^a^ | 21.54±1.36^b^ | 10.6±0.43^ab^ | 13.36±2.87^abcd^ | 8.32±0.22^cd^ | 10.49±1.32^cde^ | 0.37±0.08^c^ | 4.75±0.1^bc^ | 18±2%^b^ |
|  | 60-100cm | 67.4±5.53^a^ | 9.68±0.87^b^ | 11.95±1.05^ab^ | 14.9±0.35^abcd^ | 2.76±0.22^cd^ | 15.05±1.98^cde^ | 0.34±0.06^c^ | 4.9±0.05^bc^ | 19±2%^b^ |
| MMF | 0-20cm | 69.89±1.88^ab^ | 16.25±1.86^b^ | 14.55±4.36^abc^ | 43.64±1.31^a^ | 25.63±2.79^abcd^ | 44.26±4.56^ab^ | 1.74±0.27^ab^ | 4.59±0.15^cde^ | 20±1%^cd^ |
|  | 20-60cm | 27.65±0.43^ab^ | 7.3±0.47^b^ | 9.65±1.33^abc^ | 11.78±1.95^a^ | 11.35±0.61^abcd^ | 16.3±1.04^ab^ | 0.81±0.07^ab^ | 4.54±0.09^cde^ | 14±0%^cd^ |
|  | 60-100cm | 23.29±2.33^ab^ | 12.88±0.95^b^ | 9.27±0.77^abc^ | 10.25±1.45^a^ | 6.8±0.85^abcd^ | 16.49±3.15^ab^ | 0.76±0.07^ab^ | 4.53±0.06^cde^ | 16±0%^cd^ |
| YMF | 0-20cm | 67.4±5.53^ab^ | 49.82±0.06^a^ | 9.59±1.05^cd^ | 32.89±2.14^abc^ | 41.3±1.52^a^ | 26.1±5.48^bcde^ | 1.55±0.07^ab^ | 4.41±0.12^cd^ | 22±1%^a^ |
|  | 20-60cm | 29.52±0.43^ab^ | 44.01±0.91^a^ | 8.91±0.91^cd^ | 13.89±1.56^abc^ | 15.27±0.44^a^ | 14.25±0.75^bcde^ | 0.85±0.05^ab^ | 4.57±0.1^cd^ | 20±1%^a^ |
|  | 60-100cm | 27.4±1.35^ab^ | 31.15±1.52^a^ | 8.41±0.95^cd^ | 10.33±2.03^abc^ | 14.51±2.11^a^ | 12.89±1.72^bcde^ | 0.81±0.06^ab^ | 4.74±0.04^cd^ | 21±1%^a^ |
| OBF | 0-20cm | 35.13±0.57^a^ | 17.19±1.18^b^ | 13.8±0.71^bc^ | 7.35±1.02^d^ | 24.22±0.81^abcd^ | 11.77±0.57^de^ | 1.05±0.04^bc^ | 4.51±0.03^de^ | 22±1%^a^ |
|  | 20-60cm | 68.4±1.42^a^ | 13.23±0.41^b^ | 9.31±3.13^bc^ | 5.53±2.35^d^ | 15.01±0.87^abcd^ | 1.02±0.23^de^ | 0.56±0.06^bc^ | 4.48±0.06^de^ | 23±0%^a^ |
|  | 60-100cm | 35.5±1.8^a^ | 20.74±0.61^b^ | 8.29±1.82^bc^ | 8.79±2.3^d^ | 4.65±0.22^abcd^ | 15.15±1.94^de^ | 0.5±0.04^bc^ | 4.62±0.05^de^ | 22±1%^a^ |
| MBF | 0-20cm | 68.4±1.42^ab^ | 13.33±0.58^b^ | 14.87±1.09^ab^ | 36.61±1.31^abcd^ | 34.1±4.8^abc^ | 34.3±3.62^abcd^ | 1.63±0.12^ab^ | 4.48±0.25^b^ | 17±1%^d^ |
|  | 20-60cm | 24.04±1.66^ab^ | 10.14±0.29^b^ | 11.47±1.82^ab^ | 8.95±1.88^abcd^ | 12.23±0.39^abc^ | 11.8±1.27^abcd^ | 0.74±0.04^ab^ | 4.79±0.28^b^ | 15±0%^d^ |
|  | 60-100cm | 19.93±0.99^ab^ | 7.02±0.8^b^ | 11.23±1.65^ab^ | 7.43±0.98^abcd^ | 4.52±0.44^abc^ | 15.4±0.57^abcd^ | 0.45±0.11^ab^ | 5.04±0.28^b^ | 14±1%^d^ |
| YBF | 0-20cm | 35.5±1.8^b^ | 38.14±2.75^b^ | 12.17±0.12^bcd^ | 26.34±0.74^abcd^ | 22.47±1.2^abcd^ | 13.03±2.84^e^ | 1.24±0.08^ab^ | 4.87±0.05^a^ | 19±0%^b^ |
|  | 20-60cm | 17.56±1.14^b^ | 13.43±1.94^b^ | 10.33±2.47^bcd^ | 7.34±1.75^abcd^ | 12.23±1.09^abcd^ | 5.03±1.37^e^ | 0.69±0.09^ab^ | 4.9±0.05^a^ | 18±2%^b^ |
|  | 60-100cm | 15.19±0.87^b^ | 1.25±0.47^b^ | 7.95±0.48^bcd^ | 4.84±1.36^abcd^ | 9.83±0.58^abcd^ | 5.36±1.15^e^ | 0.76±0.02^ab^ | 5.23±0.03^a^ | 20±3%^b^ |
